# Correlation between surface chemistry and magnetism in iron nanoparticles†

**DOI:** 10.1039/d1na00258a

**Published:** 2021-06-29

**Authors:** Lorraine Haim, François Robert, Laurent Peres, Pierre Lecante, Karine Philippot, Romuald Poteau, Marc Respaud, Catherine Amiens

**Affiliations:** LCC (Laboratoire de Chimie de Coordination) BP44099, 205 route de Narbonne F-31077 Toulouse Cedex 4 France catherine.amiens@lcc-toulouse.fr; Université de Toulouse, UPS, INPT F-31077 Toulouse Cedex 4 France; CEMES (Centre d'Elaboration de Matériaux et d'Etudes Structurales), CNRS 29 rue J. Marvig F-31055 Toulouse France; LPCNO (Laboratoire de Physique et Chimie des Nano-Objets), UMR 5215 INSA, CNRS, UPS 135 Avenue de Rangueil F-31077 Toulouse Cedex 4 France romuald.poteau@univ-tlse3.fr

## Abstract

To shed light on the factors governing the stability and surface properties of iron nanoparticles, a series of iron nanoparticles has been produced by hydrogenation of two different iron amido complexes: the bis[bis(trimethylsilyl)amido] Fe(ii), [Fe(N(SiMe_3_)_2_)_2_]_2_, and the bis(diphenylamido) Fe(ii), [Fe(NPh_2_)_2_]. Nanostructured materials of bcc structure, or nanoparticles displaying average sizes below 3 nm and a polytetrahedral structure, have been obtained. Depending on the synthesis conditions, the magnetization of the nanoparticles was either significantly lower than that of bulk iron, or much higher as for clusters elaborated under high vacuum conditions. Unexpectedly, hydrogenation of aromatic groups of the ligands of the [Fe(NPh_2_)_2_] precursor has been observed in some cases. Confrontation of the experimental results with DFT calculations made on polytetrahedral Fe_91_ model clusters bearing hydrides, amido and/or amine ligands at their surface, has shown that amido ligands can play a key role in the stabilisation of the nanoparticles in solution while the hydride surface coverage governs their surface magnetic properties. This study indicates that magnetic measurements give valuable indicators of the surface properties of iron nanoparticles in this size range, and beyond, of their potential reactivity.

## Abstract 2

Amido and hydrides ligands govern stability and magnetic properties of iron nanoparticles: magnetization could be an indicator of reactivity.

## Graphical abstract

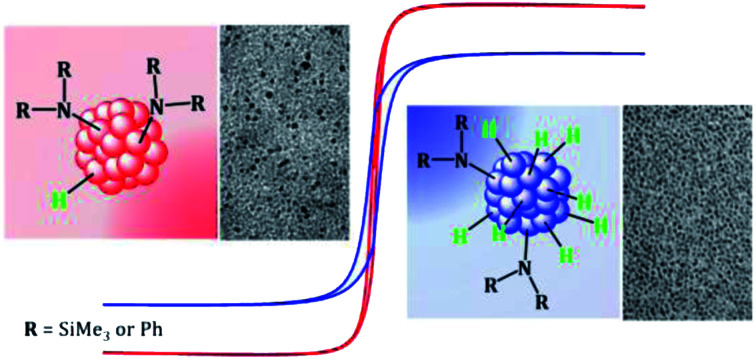

## Introduction

With the growing demand for sustainable and greener technologies, the abundant and environment friendly iron element has become one of the most attractive of the transition metals and is the focus of a large research effort. During the past few years new applications have been discovered for iron-containing materials at the nanoscale. Mostly the use of iron oxides is reported but more recently the high potential of zerovalent iron nanoparticles has also been pointed out, especially in nanomagnetism^1^ and nanocatalysis,^2,3^ despite the difficulty of their handling. This suggests that controlling the size, shape and surface state of iron nanoparticles (NPs) is a pressing issue to allow the development of *e.g.* new powerful magnets or selective catalytic processes. As for any synthesis, the synthesis of NPs starts from the choice of a proper starting material and, concerning iron(0) NPs, finding an appropriate iron source is still an active field of research. As well, identification of the key parameters that govern the iron(0) NPs stabilization during their growth, their final surface state and magnetic properties remains a challenge. Given the extreme air and water sensitivity of iron, especially at the nanoscale, working in air tight conditions and in dry organic solvents is mandatory which points to the use of metal organic complexes as iron sources. Three main classes of metal–organic complexes have been investigated with their specific advantages and drawbacks: olefinic and aromatic derivatives (relatively inert),^4,5^ carbonyl derivatives (easily accessible but risk of carbon and oxygen contamination),^6–9^ and the bis[bis(trimethylsilyl)amido] Fe(ii) complex [Fe(N(SiMe_3_)_2_)_2_]_2_ (commercially available but expensive and difficult to handle). When exposed to an hydrogen atmosphere, amido complexes are expected to release the corresponding amine in the course of the reaction, a type of ligand often used to control the stability and morphology of NPs in colloidal solutions.^10–13^ [Fe(N(SiMe_3_)_2_)_2_]_2_ has been reported by our group and others to afford iron NPs of controlled size and shape, and deprived of any surface oxidation, affording systems particularly adapted to investigate surface magnetism at the nano-scale.^14–19^ Surface effects are best observed on NPs which display a high enough proportion of surface atoms, namely those having a size below 3 nm.^20,21^ In this size range, analysis of the data published so far on iron NPs prepared by hydrogenation of [Fe(N(SiMe_3_)_2_)_2_]_2_, shows that NPs of identical size and structure can display different magnetic features.^14,17^ This clearly points to the influence of adsorbed surface species, which vary depending on the synthesis conditions, and also suggests that magnetic measurements could be good indicators of surface properties. In catalysis this would be helpful to predict the potential reactivity of new iron(0) NPs and beyond, help understand their catalytic performances. The NPs prepared by hydrogenation of [Fe(N(SiMe_3_)_2_)_2_]_2_ were demonstrated to display surface hydrides and to be efficient hydrogenation catalysts.^22^ As a consequence, the effect of surface hydrides on magnetization can be questioned. From a theoretical point of view, if surface magnetism depletion was found upon adsorption of H on Fe(*hkl*) slabs, it still remained above the value of bulk bcc iron, and no clear influence on magnetism is expected from the majority of ligands (like amines or carboxylic acids, or adsorbed Cl atoms).^23,24^

This motivated us to set up a dual experimental and theoretical investigation to try and answer these questions. Especially we looked for an alternative amido complex to avoid any side reaction that may originate from the bis(trimethylsilyl)amido ligand of [Fe(N(SiMe_3_)_2_)_2_]_2_.^25,26^ Among the few non silylated amido complexes of Fe(ii) reported so far in the literature, we chose the bis(diphenylamido) iron(ii) complex [Fe(NPh_2_)_2_] for its stability and crystallinity which facilitated its purification, characterization and handling.^27^ We here report on the synthesis of a set of iron(0) NPs with varying chemical environments, the interaction of which with the surface has been theoretically studied. Given the size and the chemical complexity of the experimental systems, our theoretical contribution does not aim at reproducing experimental data, but at providing trends. Confrontation between magnetic measurements and the trends deduced from the theoretical calculations shed light on the key parameters that govern stability and magnetization values in these systems.

## Results

### Synthesis and characterisation of the iron NPs

**Scheme 1:**
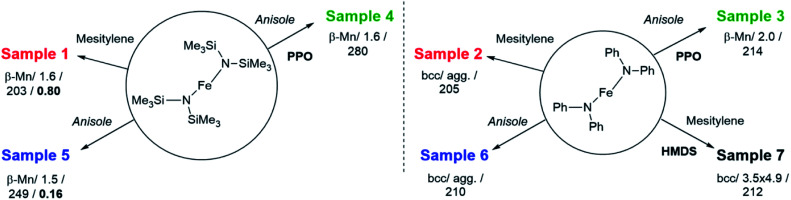
Synthetic pathways for the samples synthesized in this work. Duration (48 h), temperature (150 °C) and hydrogen pressure (3 bar) were identical for all samples. PPO = polydimethylphenylene oxide, HMDS = hexamethyldisilazane. Structure/size (nm)/magnetization (Am^2^ kg_Fe_^−1^)/hydride coverage (number of hydrides per Fe surface atom) are reported below the name of each sample.

First, hydrogenation of [Fe[N(SiMe_3_)_2_]_2_]_2_ in mesitylene (Scheme 1, Table S1 and Scheme S1†) was carried out to confirm the results described by Lacroix *et al.*^17^ Iron NPs, easy to disperse in organic solvents, were obtained as a black powder (sample 1). Wide-angle X-ray scattering (WAXS) data (Fig. S1 and S2†) and transmission electronic microscopy (TEM) images (Fig. S3†) confirmed their polytetrahedral β-Mn structure and average size (1.6 ± 0.4 nm). Titration of surface hydrides led to a value of 0.80 ± 0.03 H per surface iron atom.

To try and evidence the possible effect of silylated by-products on the magnetic properties of the iron NPs, a new, silicon free amido complex was used for comparison purposes: [Fe(NPh_2_)_2_]_2_. This bis(diphenylamido) iron(ii) complex was prepared by reacting FeBr_2_ and LiNPh_2_ as described by Olmstead *et al.*^27^ or by metathesis between [Fe[N(SiMe_3_)_2_]_2_]_2_ and diphenylamine (DPA) following a procedure adapted from the work of Merrill *et al.*^28^ Both methods led to dark red crystals in respectively moderate to good yields from which the complex [Fe(NPh_2_)_2_]_2_ was unambiguously identified by elemental analysis, nuclear magnetic resonance and infra-red spectroscopy.

**Fig. 1:**
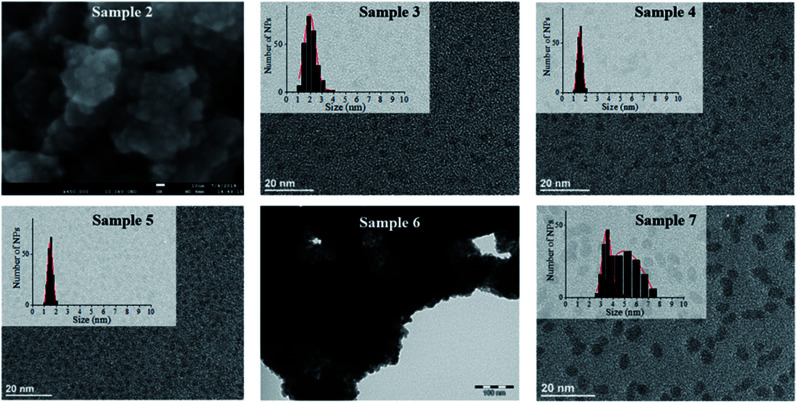
Clockwise from top left: SEM analysis of sample 2 (scale bar = 10 nm), TEM analysis of sample 3 (scale bar = 20 nm), sample 4 (scale bar = 20 nm), sample 5 (scale bar = 20 nm), sample 6 (scale bar = 100 nm) and sample 7 (scale bar = 20 nm).

Hydrogenation of [Fe(NPh_2_)_2_]_2_ was first attempted in the same conditions as for sample 1 to allow for a direct comparison of the results (sample 2, Schemes 1 and S1†). This precursor readily decomposed at 150 °C in mesitylene affording an insoluble black powder that was easily collected on the magnetic stirring bar and a colourless transparent solution. This solution was analysed by gas chromatography coupled to mass spectrometry (GC-MS) to identify the by-products issued from the diphenylamido ligand (Fig. S4†). Three compounds could be evidenced: diphenylamine, cyclohexylphenylamine and dicyclohexylamine. Analysis of the powder by inductively coupled plasma – optical emission spectroscopy (ICP-OES) indicated a very high iron content (96.7 w%). The morphology of the powder was investigated by scanning electron microscopy (SEM) (Fig. 1, top left and S5†). Images collected at different magnifications showed a multiscale structuring of the powder from micron large flakes down to grains no larger than 10 nm and relatively homogeneous in size. This analysis was confirmed by the TEM investigation of a few flakes that could be dispersed in toluene, drop casted on a TEM grid and were thin enough to be observed (Fig. S6†). The structural WAXS analysis of sample 2 (Fig. S1 and S2†) indicated a bcc structure with cell parameters identical to those of the reference bcc-Fe bulk structure.

To limit the growth and coalescence of the NPs, we used a polymeric matrix, choosing one that had already been reported for the stabilization of ultrasmall iron and cobalt NPs:^15,29,30^ poly-(2,6-dimethyl-1,4-phenylene oxide) (PPO) keeping all reaction parameters identical but the solvent. Indeed, as PPO is not soluble in mesitylene, a more polar aromatic solvent, anisole, was used instead (sample 3, Schemes 1 and S1†).

The metal over polymer ratio was fixed to 5 w%. At the end of the reaction, drop casting of the black colloidal solution obtained on a TEM grid allowed the observation of well dispersed NPs with an average size of 2.0 nm ± 0.9 nm (Fig. 1). WAXS data were collected and studied in direct and reciprocal spaces (Fig. S1 and S2,† respectively). They indicated a β-Mn structure, as frequently observed for iron NPs with sizes below 5 nm.^14,17^ For comparison purposes, the same procedure was reproduced, this time using [Fe[N(SiMe_3_)_2_]_2_]_2_ as a precursor. Indeed experiments previously reported in the literature were carried out at lower temperature (110 °C) and for a shorter reaction time (12 h) precluding any direct comparison.^15^ The black shiny material recovered (sample 4, Schemes 1 and S1†) was analyzed according to the same procedures and methods than sample 3. TEM images (Fig. 1) showed well dispersed NPs with an average diameter of 1.6 ± 0.6 nm. As for sample 1, WAXS investigations indicated a β-Mn structure (Fig. S1 and S2†).

To investigate any possible solvent effect, iron NPs were also synthesized by hydrogenation of [Fe[N(SiMe_3_)_2_]_2_]_2_ in anisole instead of mesitylene (sample 5, Schemes 1 and S1†). The NPs obtained displayed an average diameter of 1.5 ± 0.4 nm (Fig. 1), and a β-Mn structure (Fig. S1 and S2†). Comparatively to sample 1, a much lower quantity of surface hydrides was found for this sample, namely 0.16 ± 0.03 H per surface iron atom.

In comparison, in the absence of PPO, hydrogenation of the bis(diphenylamido) iron(ii) complex in anisole (sample 6, Schemes 1 and S1†) only led to a black magnetic powder consisting in strongly aggregated NPs of bcc structure as evidenced by WAXS (see Fig. S1 and S2†). This is similar to what was observed following its hydrogenation in mesitylene in the absence of polymer (sample 2). These NPs could not be dispersed preventing any size determination by TEM. Fig. 1 shows a typical TEM image of a large ensemble.

Lastly, to investigate the potential role of hexamethyldisilazane (HMDS) during the synthesis (sample 7, Scheme 1), hydrogenation of the bis(diphenylamido) iron(ii) complex was also performed in the same conditions as for sample 2 but in the presence of 2 molar equivalents of HMDS per iron atom. This led to a black colloidal solution from which GC-MS analysis indicated that DPA had been fully hydrogenated (Fig. S7†). Traces of 1,3,5-trimethylcyclohexane were also detected indicating that the solvent was also subject to hydrogenation. TEM analysis showed well dispersed prolate NPs with 4.9 nm in length and 3.5 nm in width (Fig. 1). WAXS analysis revealed a bcc structure in this case.

It is noteworthy that when the coherence length of the crystallites could be obtained by analysis of the RDF extracted from the WAXS data (Fig. S2†), it was in good agreement with the mean diameter determined from TEM images indicating structurally well-defined objects (see Table 1). The slight discrepancy observed for sample 7 might suggest polycrystalline NPs. Also, no trace of oxidation could be detected by WAXS for any of the seven samples.

**Table 1:** Main magnetic data extracted from the VSM analysis of samples 1–7, mean diameters (*d*) determined either from magnetic data analysis or from TEM images and coherence length (*λ*_c_) extracted from their WAXS RDF (see Fig. S2) (agg.) indicates the presence of large aggregates of nanoparticles

Sample	*M*_s_ aa (Am^2^ kg_Fe_−1) ± 5%/*μ*_Fe_ (*μ*_B_)	*T*_B_ bb (K) ± 0.1	*d* cc (nm)	*K*_eff_ cc (J m^−3^) ± 10%	*d* (nm) from TEM (±0.1 nm)	*λ* c (nm) WAXS (±0.1 nm)
1	203^**^/2.03	—	1.5^##^	6.3 × 10^5##^	1.6	1.5
2	205^**^/2.05	>300	—	—	≤10 nm (agg.)	>6
3	214^*^/2.14	12.2^&^	2.2^#^	3.8 × 10^5#^	2.0	1.6
4	280^*^/2.80	11.3^&^	1.7^#^	7.9 × 10^5#^	1.6	1.5
5	249^**^/2.49	9.1^&&^	1.7^##^	8 × 10^5##^	1.5	1.5
6	210^**^/2.10	>300	—	—	(agg.)	>6
7	212^**^/2.12	—	—	—	4.9 (length)	3.0
					3.5 (width)	

a Measured at 2.5 (*) or 5 K (**).

b From FZC/FC curves recorded at 10 mT (&) or 2.5 mT (&&).

c From the fit of ZFCFC (#) or from M (H) curves in superparamagnetic regime (##).

The magnetic properties of these samples were thoroughly studied (see Section 7 in ESI†) and the main data extracted from the hysteresis cycles and fit of the zero field cooled/field cooled (ZFC/FC) curves are reported in Table 1.

All samples displayed the ferromagnetic behaviour characteristic of metallic iron; especially no trace of iron oxide could be detected. Samples 2 and 6 displayed a hysteretic behaviour at r.t. as expected for nanostructured iron powders of bcc structure, with saturation magnetization values reaching respectively 205 ± 10 and 210 ± 11 Am^2^ kg_Fe_^−1^ while samples 1, 3–5 and 7 displayed the characteristic superparamagnetic transition of iron NPs with mean sizes below 5 nm. The mean sizes determined from the fit of the experimental magnetic data where all in good to excellent agreement with those determined from TEM images (see Table 1), and with the coherence length of the crystallites (Table 1 and Fig. S2†). Interestingly samples 3 and 4 exhibited saturation magnetization values largely above that of bulk iron (respectively 280 ± 14 and 249 ± 13 Am^2^ kg_Fe_^−1^), while those determined for samples 1, 3 and 7 were all below that of bulk iron (2.22 *μ*_B_ = 222 Am^2^ kg_Fe_^−1^ at 5 K). Slight variations in the effective magnetic anisotropy values were also observed in the range 3.8–7.9 × 10^5^ J m^−3^ (±10%).

The results obtained from this comparative study evidenced the crucial effect of the precursor on the final properties of the nanomaterials, and the key role of HMDS on the stabilization of ultrasmall NPs. Interestingly, most of these systems displayed NPs with average sizes of *ca.* 1.6 nm, a size at which surface magnetism is the main contributor to the magnetic properties. However, only two synthesis protocols led to samples with the enhanced magnetization expected in this size range^31^ while the others led to samples with bulk-like magnetization. It is noteworthy that given the air sensitivity of the nanomaterials, each synthesis was repeated at least 2 times, and reproduced by different operators, to rule out any artefact that might have arisen due to adventitious oxidation. The variations in saturation magnetization between samples, as reported herein, were repeatedly observed. This, as well as the fact that no oxide traces could be detected either by structural and magnetic measurements, points to the implication of surface chemical species.

To go further, theoretical calculations were thus performed in order to determine how HMDS, DPA, or H may interact with the iron(0) NPs surface, and their influence on the magnetic properties.

### Theoretical calculations

DFT investigations were performed to assess the possible effect of the geometry of the iron NPs or of their surface composition on their magnetic properties. The structural, energy and magnetic moments of bulk bcc iron at the chosen DFT level of calculation (see Experimental section for details) are given in Table S2.† As well known,^32^ whereas in the case of iron cohesive energies are overestimated, the lattice constant and magnetization are both very accurate. The NPs synthesized display mean sizes in the range 1.5 to 2.0 nm, which corresponds to approximately 200 to 300 metal atoms, a somewhat too large size for DFT calculations. Given also the necessity to stabilize the surface with numerous hydrides and ligands, more affordable 91 iron atoms models were built as presented in Fig. S15.†

Yet, ferromagnetism in iron clusters and nanoclusters is size-dependent,^33,34^ and the magnetization of these models was expected to be higher than the experimental values reported in this study. But relevant trends could be extrapolated from these models to larger NPs.

**Fig. 2:**
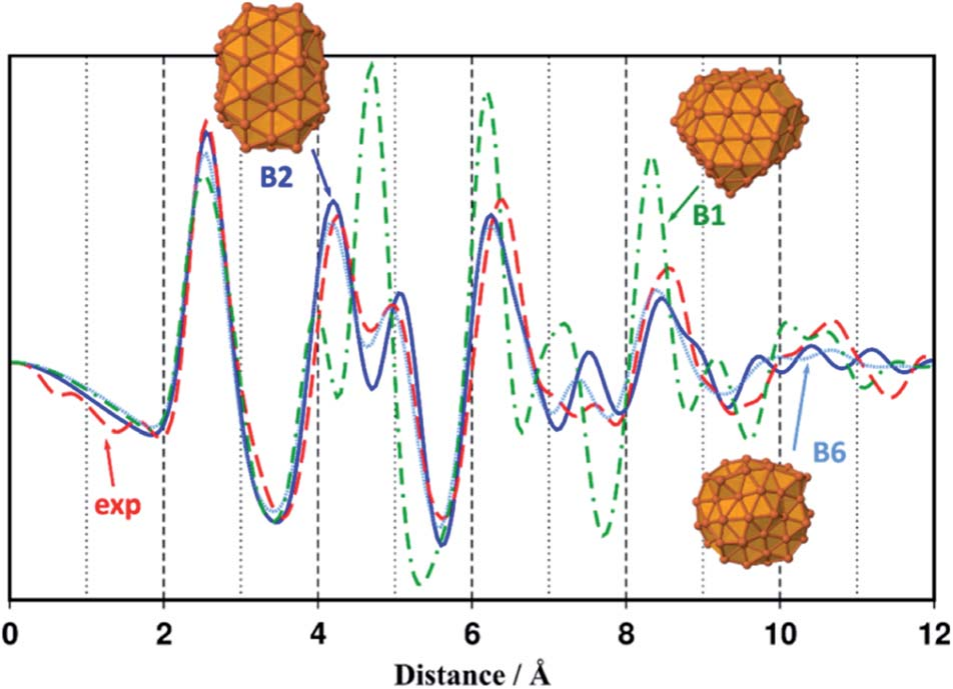
Simulated RDF profile for the rhombic dodecahedron B1–Fe_91_ (green dashed dotted line) and polytetrahedral B2–Fe_91_ and B6–Fe_91_ clusters (blue plain line and light blue dotted line), compared with the experimental RDF profile of sample 1 (red dashed line). Geometries, magnetic moments and cohesive energies of all Fe_91_ isomers considered in this study (B1–B7), are reported in Fig. S15.†

A comparison of relative energies and magnetic properties for seven 91-atoms isomers is reported in Fig. S15.† All isomers have magnetic moments per atom close to 2.8 *μ*_B_. The lowest energy isomer (B1) was made from a parent 175-atoms bcc rhombic dodecahedron (RDD), sliced down to 91 atoms. Its cohesive energy is 3.97 eV (bulk: 4.85 eV) and as expected its magnetization is higher than the bulk one (2.73 *μ*_B_*vs.* 2.22 *μ*_B_). However, as shown in Fig. 2, its RDF profile could not fit the WAXS data collected from samples 1 and 3–5. Among the other isomers, the Finnis–Sinclair Fe_91_ cluster of Wales and co-workers^35^ (B2) is *ca.* 24 kcal mol^−1^ less stable that the RDD-derived one. Its cohesive energy is 3.95 eV and its magnetic moment per atom is 2.82 *μ*_B_ far above that of bulk iron (2.22 *μ*_B_). It is a polytetrahedral compound which RDF profile fits the WAXS data collected from samples 1 and 3–5 (see Fig. 2, the discrepancy between intensities is related to their difference in size). The RDF profile of a β-Mn spheroidal cluster also optimized at the DFT level of calculation is reported in Fig. 2 (isomer B6). It is very similar to the experimental and B2 profiles. Given that it lays 66 kcal mol^−1^ above B2, all studies were then performed on the more stable slightly prolate B2 model, which presents 65 surface atoms and diameters of *ca.* 1.0 nm and 1.2 nm for its minor and major axis, respectively.

**Fig. 3:**
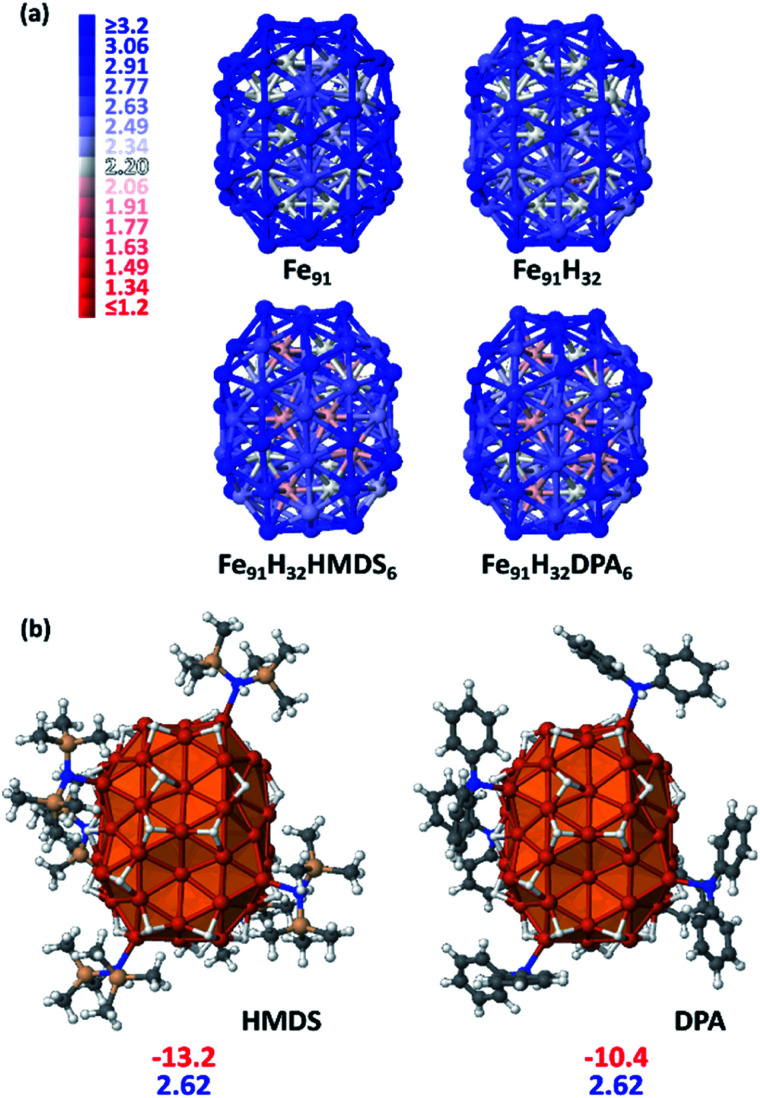
(a) Magnetization colour maps of B2–Fe_91_ models with various surface compositions (the colour scale, between 1.2 *μ*_B_ and 3.2 *μ*_B_, is given on the left). Magnetic moments of light red core atoms in HMDS- and DPA-protected Fe_91_H_32_NPs are *ca.* 2.10 *μ*_B_, instead of *ca.* 2.20 *μ*_B_ in Fe_91_ and 2.15 *μ*_B_ in Fe_91_H_32_. (b) B2–Fe_91_ model with *τ*_surf_ = 0.5 and 6 surface stabilizers. First line: adsorption energy per HMDS or DPA ligand (in kcal mol^−1^); second line: magnetic moment per iron atom (in *μ*_B_).

Most iron atoms in the core of B2 have a magnetic moment (2.20 *μ*_B_) close to that of bulk iron, whereas it is slightly higher for a couple of them (*ca.* [2.5–2.7] *μ*_B_), as well observed in the magnetization colour map given Fig. 3a. Interestingly, this difference is related to their coordination number (CN) and local environment. It is shown in Fig. S16† in terms of coordination polyhedra: all atoms with a magnetic moment of 2.20 *μ*_B_ have a local icosahedral environment (CN: 12). Such environment-related behaviour was also observed for a hypothetical β-Mn-type phase of bulk iron (see Table S2 and Fig. S17†). The high magnetization of this Fe_91_ cluster stems from surface atoms with local magnetic moments close to 3.2 *μ*_B_.

The first question addressed by this DFT study was the possible effect of the amines on magnetic properties. Do they quench or increase magnetization? Given previous experimental quantitative analysis^22^ and the results reported herein from samples 1 and 5 at first an intermediate hydride surface coverage (*τ*_surf_) value of 0.5 (*i.e.* 0.5 H per surface Fe atom) was randomly grafted on three-fold coordination (*μ*_3_) surface sites. This led to 32 hydrides as B2 has 65 surface atoms. The average dissociative adsorption energy per hydride is −14.6 kcal mol^−1^. The 32 hydrides significantly decrease the average magnetic moment per atom by 0.18 *μ*_B_ (2.64 *vs.* 2.82 *μ*_B_). Whereas they do not impact the magnetic properties of core atoms, they lower the magnetization of atoms on which they are coordinated by *ca.* [0.2–0.4] *μ*_B_/Fe_surf_. This can be seen on the magnetization colour maps of B2–Fe_91_ and B2–Fe_91_H_32_ plotted in Fig. 3a. Then, 6 HMDS or DPA ligands were coordinated on the B2–Fe_91_H_32_ model, on the same surface sites for better comparison. A study of the equilibrium composition of H and ligands was beyond the scope of this theoretical study. But six is large enough to reveal the effect of the ligands on the magnetization of the metal core (see Fig. 3b), although it is likely that the surface of this nanocluster can afford more ligands. The adsorption energy per ligand is moderate, of the order of magnitude of the dissociative adsorption energy of hydrides (HMDS: −13.2 kcal mol^−1^ and DPA: −10.4 kcal mol^−1^). Both sets of 6 ligands involve the same slight decrease of the magnetic moment per atom, by 0.02 *μ*_B_ (2.62 *μ*_B_ after adsorption of 6 HMDS or DPA ligands *vs.* 2.64 *μ*_B_ for B2–Fe_91_H_32_). Contrarily to hydrides the effect of which is mainly observed on the coordination site, coordination of 6 HMDS or DPA ligands induces a small decrease of the magnetic moment of some core atoms below the bulk value (light red atoms in Fig. 2a). It is noteworthy that adsorption energies and magnetic moments should not differ much between DPA, cyclohexylphenylamine and dicyclohexylamine, the main by-products of the hydrogenation reaction.

In summary, with such surface composition it is the hydrides that have the greatest influence on magnetization, whilst HDMS or DPA have a similar and moderate impact (−0.02 *μ*_B_) *e.g.* the average magnetic moment is lowered by 0.2 *μ*_B_ between the naked Fe_91_ cluster and the HMDS-protected B2–Fe_91_H_32_ cluster reported in Fig. 3 (2.82 *vs.* 2.62 *μ*_B_).

Considering that the amine ligands are in their amido form in the iron complexes used to generate the NPs, the magnetization of the Fe_91_H_32_ clusters stabilized by amido forms of DPA and HMDS were also determined. The results are reported in Fig. S19.† There is almost no effect on the magnetic properties. It is however noteworthy that whereas adsorption energies of HMDS and its amido counterpart are similar (the latter being only slightly more stable at the surface), from a thermodynamics point of view the amido form of DPA is significantly more stable on the surface, by ∼5 kcal mol^−1^ per ligand, than the amine itself. This energy difference is large enough to support the idea that DPA lays on the surface as σ-donor diphenylamido ligands. Yet, π-interaction of phenyl groups with the surface are likely to occur, but it was not observed on the chosen coordination sites.

We further investigated the possible adsorption of solvent molecules and their eventual effect on the global magnetization of the Fe_91_H_32_ cluster. It was found that with an adsorption energy per molecule of −16.7 kcal mol^−1^, anisole which is π-coordinated on the surface could compete on iron sites with the stabilizing ligands. Furthermore, they tend to reduce the magnetic moment by 0.07 *μ*_B_ per iron atom (see also Fig. S19†). The same trend is expected for mesitylene.

**Fig. 4:**
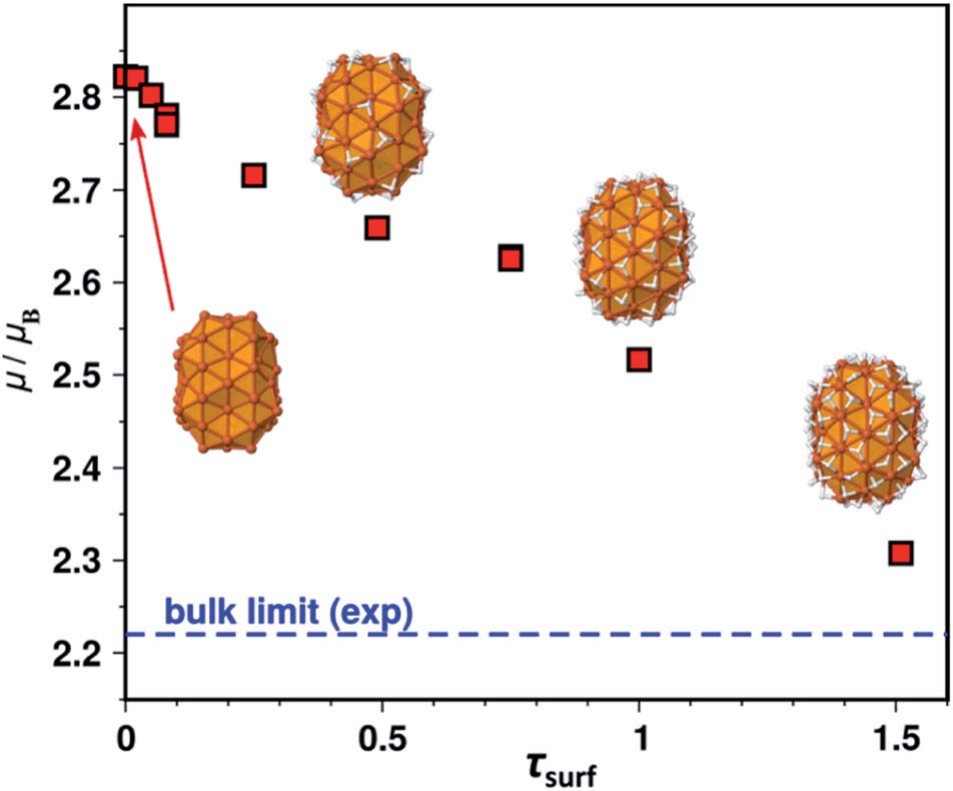
Evolution of the magnetic moment per Fe atom of B2–Fe_91_H_*n*_ as a function of *τ*_surf_.

We shall now examine the relationship between the number of surface hydrides and the magnetic moment. Several surface hydride coverages were considered on B2, yielding a series of Fe_91_H_*n*_ species (*n* = 1–98, *i.e. τ*_surf_ = 0.02–1.51 given the model has 65 surface atoms). The magnetic moment per atom is reported in Fig. 4 as a function of *τ*_surf_. It decreases regularly from the bare NP (on the left) to its hydride-saturated counterpart (on the right). Two cases were considered for *τ*_surf_ = 0.49, namely 32 surface hydrides, or 29 surface hydrides and 3 hydrides in tetrahedral sites of the core. Both exhibit the same magnetic moment, 2.64 *μ*_B_. The 0.3 *μ*_B_ decrease observed between the bare NP and the (*τ*_surf_ = 1.0)-NP (*i.e.* Fe_91_H_65_) is an interesting point of reference.

## Discussion

### Stabilization of iron NPs

The two precursors used to prepare the iron NPs reported herein led to very different nanomaterials. Especially, the silylated derivative [Fe[N(SiMe_3_)_2_]_2_]_2_ afforded well dispersed iron NPs of very small sizes even in the absence of external stabilizer (samples 1 and 5) while in such conditions only large, ill-stabilized NPs were obtained from the bis(diphenylamido) iron (ii) complex (samples 2 and 6). When exposed to a hydrogen atmosphere, amido complexes are expected to release the corresponding amine in the course of the reaction, a class of ligands often used in NPs synthesis and stabilization.^10–13^ Contrarily coordination of amido ligands at the surface of NPs was only seldom discussed.^36^ Here, DFT calculations showed that: (1) adsorption of the diphenylamido ligand is significantly more favourable on the iron surface than that of DPA, (2) adsorption of the bis(trimethylsilyl) amido ligand is only slightly more favoured than that of HMDS and (3) adsorption energies of all amines are comparable. Based on thermodynamics considerations, the [Fe(NPh_2_)_2_] complex should easily afford ultrasmall iron NPs stabilized by diphenylamido ligands. This is in striking contrast with the experimental results (see samples 2 and 6) as only large, ill-stabilized NPs were formed when using this precursor in the absence of PPO. If persistence of amido ligands at the surface of the NPs cannot be fully discarded, confrontation between experimental data and theoretical calculations suggest that kinetics effect could be the reason for the larger size of the NPs formed. It suggests that decomposing the bis(diphenylamido) iron (ii) complex in milder conditions could afford NPs of smaller size.

Well dispersed and well defined NPs of much smaller size (1.6 ± 0.2 nm) were obtained upon hydrogenation of [Fe(N(SiMe_3_)_2_)_2_]_2_ in the same conditions of temperature, pressure and reaction time, in good agreement with previous literature data.^18^ Interestingly, when hexadecylamine was introduced in the medium at the beginning of the reaction, only ill defined NPs were obtained.^37^ In comparison, adding HMDS to the reacting medium with [Fe(NPh_2_)_2_]_2_ (sample 7) limited the coalescence of the NPs whilst in the sole presence of DPA and its hydrogenated by-products only large polycrystalline objects were obtained. This suggests that DPA and derived amines interact less efficiently with the iron surface than HMDS and its amido counterpart. However, as DFT calculations showed that the adsorption energies of all these amines are not significantly different, stabilization by the bis(trimethylsilyl) amido ligand should not be discarded and is supported by the more acidic character of HMDS in comparison to that of the alkylamines or even phenylcyclohexylamine.^38^

Ultrafine iron NPs were reported to efficiently catalyse the hydrogenation of alkenes and alkynes^22,39^ but to our knowledge, no hydrogenation of aromatic rings was reported so far. Detection of cyclohexylphenylamine and dicyclohexylamine by GC-MS analysis in samples 2 and 7 was at first rather unexpected. Note that an excess of H_2_ was still present in the reactor at the end of the synthesis but that full conversion into dicyclohexylamine was seldom reached. This indicates that the species responsible for the hydrogenation of DPA was no more active at the end of the synthesis. We can thus conclude that the *ca.* 10 nm large NPs observed in the nanostructured powder recovered at the end of the synthesis are not the active species. It suggests that decomposing the bis(diphenylamido) iron (ii) complex in milder conditions could afford NPs of interest for catalytic applications.

### Relation between surface species and magnetic properties

To discuss the effect of the surface species on the magnetic properties of iron at the nanoscale, we shall here focus on samples with sizes below 5 nm (samples 1, 3–5 and 7) which are the best adapted to point a correlation between surface state and magnetization values. Moreover, the atomic packing was that of β-Mn in all of these samples as very often observed for NPs in this size range,^14,30,40^ with similar inter-atomic distances, affording an opportunity to directly correlate surface state and magnetic properties. DFT calculations showed that adsorption of aromatic molecules on the surface may induce a drop in surface magnetization. As all samples were prepared in aromatic solvents, we will first assume that the differences observed are from a different origin. The results reported in Table 1 and on Scheme 1 show that the two precursors give very different results, especially concerning their saturation magnetizations, and effective anisotropy. Samples 3 and 7, prepared from [Fe(NPh_2_)_2_], both display magnetization values below that of bulk iron. Based on the results from DFT calculations, we assume that these low magnetization values are due to the presence of hydrides and diphenylamido ligands at the surface of the NPs. Concerning the NPs prepared from [Fe(N(SiMe_3_)_2_)_2_]_2_ (samples 1, 4 and 5) important variations are observed despite identical size and structure, once again pointing to surface chemistry effect. From theoretical calculations and measurements made on bare iron clusters prepared in the gas phase, a magnetic moment per atom of circa 2.8 *μ*_B_ is expected for 1.6 nm large iron clusters.^31,33,41^ Here sample 4 presents a saturation magnetization value in perfect agreement with these previous reports. However, sample 1 presents a much lower magnetization value. Supposing that the polymeric matrix provides only steric stabilisation *i.e.* that the number of chemical bonds that it forms with the surface is very limited (and thus negligible) due to steric hindrance, we explored the possible effect of the amido ligand or corresponding amine resulting from the precursor, as well as that of hydrides. DFT calculations carried out on model clusters revealed that local magnetic moments are very sensitive to hydrides adsorption in comparison to amine/amido adsorbates. Moreover, due to their steric hindrance, it is believed that the amine/amido ligands represent only a limited fraction of the adsorbed species, contrarily to hydrides. We thus inferred that the variation in hydride coverage from one sample to the other was the principal factor governing the magnetization of the NPs. Hydride titration was thus carried out for two typical samples, samples 1 and 5, which differ only by the solvent used for the synthesis. In perfect agreement with the above hypothesis, sample 1 which presented the lowest saturation magnetization of the two (2.03 ± 0.1 *vs.* 2.49 ± 0.12 *μ*_B_) also displayed the highest hydride per surface iron atom ratio (0.80 *vs.* 0.16).

Moreover, the iron NPs prepared from [Fe(N(SiMe_3_)_2_)_2_]_2_ in the presence of PPO (sample 4), a stabilizer that can be considered as inert towards the metal surface, showed different magnetic properties that can be attributed to a different surface chemistry induced by the nature of the iron precursor. Indeed, the high magnetization values recorded for sample 4 indicates that the quantity of ligands, and especially hydrides, is negligible at the surface of the NPs. This suggests that PPO either limited the presence of hydrides or was able to scavenge hydrides from the surface, possibly being partially hydrogenated in the process.

Concerning sample 3, also prepared in the PPO matrix but using [Fe(NPh_2_)_2_], its comparatively low magnetization indicates that ligands are still present at the surface, such as the diphenylamido ligand which was shown by DFT calculation to present the highest adsorption energy. This suggests that hydrogenation of DPA into the more weakly interacting cyclohexyl derivatives did not take place. Unfortunately, analysis of the solution by GC-MS could not be performed due to the presence of the polymer and it is not possible to conclude on these hypotheses. However, these results support well the idea that the nature of the iron precursor plays a key role in the control of the surface chemistry and its influence on the magnetic properties of the iron NPs here described.

Note that the effective magnetic anisotropy was also influenced. As a general tendency, it increased with size reduction and enhanced magnetism. A more refined analysis would require theoretical calculations including spin orbit coupling effects.

## Experimental

### Computational details

#### DFT calculations of metal nanoclusters

Software: Vienna *ab initio* simulation package, VASP;^43,44^ spin polarized DFT; exchange-correlation potential approximated by the generalized gradient approach proposed by Perdew, Burke, and Ernzerhof (PBE);^45^ projector augmented waves (PAW) full-potential reconstruction;^46,47^ PAW data sets for Fe treating the (*n* − 1)*d* and ns states (*i.e.* 8 valence electrons); kinetic energy cutoff: 500 eV; *Γ*-centered calculations;^48^ Gaussian smearing (*σ*) of 0.02 eV width, energies being therefore extrapolated for *σ* = 0.00 eV; supercell size: 30.5 × 30.75 × 31 Å^3^ (ensures a vacuum space of *ca.* 18 to 20 Å between periodic images of Fe_91_H_*n*_ metal clusters, and of 10 to 12 Å in the case of Fe_91_H_*n*_L_6_ compounds, with L = HMDS or DPA); atoms positions were optimized until the criterion of the residual forces on any direction being less than 0.02 eV Å^−1^ was met. Magnetic moments are as usually calculated as the expectation value of the total spin angular momentum, *i.e. μ*_B_(*n*_↑_ − *n*_↓_).

#### DFT calculations of bulk properties

Also calculated with VASP, at the same level of theory (*i.e.* PBE functional). bcc: (20 × 20 × 20) *Γ*-centered *k*-point grids for optimization and energy calculations; β-Mn type (polytetrahedral): (6 × 6 × 6). Optimization: Gaussian smearing of 0.02 eV width for the partial occupancies; forces less than 0.02 eV Å^−1^. Single-point calculation of magnetization and energy properties at optimized geometries, using the tetrahedron method proposed by Blöchl for the *k*-space integration.^49^ The hypothetical “β-Mn” phase of iron has been adapted from β-Mn cif coordinates. Both bcc and β-Mn lattice constants were determined by fitting the volume dependence of the static lattice energy to a Murnaghan equation of state.^50^

#### Analysis of magnetic moments per atom

Calculated after projection of the PAW wavefunction in a minimal Slater-type orbital basis set. It was achieved with the Lobster software, using the pbeVASP-fit basis set.^51–53^ Charge spilling (a criterion that assesses the quality of the projection) systematically lower than 1.0%; it involves that the sum of atomic magnetic moments is very close to the VASP global magnetization. Magnetization colour maps made with Jmol^54^ (dark blue: 3.2 *μ*_B_; white: 2.2 *μ*_B_, *i.e.* bulk bcc value; dark red 1.2 *μ*_B_).

#### Miscellaneous

3D coordinates of some bare and hydrogen-covered clusters as well as theoretical RDF profiles generated with the in-house polyhedra and NPManip softwares.^55^

Adsorption energies (* stands for adsorbed)*E*_ads_(H) = 1/*n*[*E*(*n*H*) − *E*(NP) − *n*/2*E*(H_2_)]*E*_ads_(H) = 1/*n*[*E*(*n*LH*) − *E*(NP) − *nE*(LH)]

for amido fragments*E*_ads_(L) = 1/*n*[*E*(*n*L*, *n*H*) − *E*(NP) − *nE*(LH)]

Cohesive energies*E*_coh_(L) = 1/*N*[*E*_unit cell_ − *NE*_at_]

### Characterization techniques

Iron content in the samples was determined by inductively coupled plasma-optical emission spectrometry (ICP-OES, PerkinElmer Optima 2100 DV ICP), after digesting the samples into a mixture of HNO_3_ : HCl (1 : 3 ratio v/v) and diluting them with ultrapure water.

C, H and N contents were determined on a ICAP 7600ICP-OES analyser (Thermoscientific) on a PerkinElmer 2400 série II analyser (Thermoscientific). Results reported herein are the average of two independent measurements.

FTIR spectra were recorded in ATR mode on a Bruker Alpha FT-IR spectrophotometer placed in a glove box. Data are reported in wave numbers (cm^−1^) with (s) (m) and (w) indicating respectively strong, medium and weak absorptions.

^1^H NMR spectra were obtained on a Bruker Avance 400 spectrometer, ATMA observation BB (19F–15N) measuring head, decoupling. ^1^H, diam. 5 mm at *T* = 293 K, SampleXpress sample changer with 60 positions. Chemical shifts are reported in parts per million (ppm) downfield from tetramethylsilane and are referenced to the residual solvent resonance as the internal standard.

GC-MS analysis were carried out on a GC-MS-QP2010 Ultra instrument equipped with a flame ionization detector and a Zebron ZB-5MSplus column (30 mL × 0.25 mm ID × 0.25 μm df) 5% polysilarylene – 95% polydimethylsiloxane. Helium was used as carrier gas. Analysis was performed using a temperature set from 30 to 250 °C (15 °C min^−1^). Samples were diluted in dichloromethane.

Structural powder characterizations were performed by Wide Angle X-ray Scattering (WAXS). The fine powder was introduced into a thin walled Lindemann capillary of 1 mm diameter subsequently sealed under argon. The samples were irradiated with graphite-monochromated molybdenum Kα radiation (0.071069 nm). The scattered intensity was recorded by a dedicated two-axis diffractometer equipped with a high energy resolution solid-state detector allowing for removal of the fluorescence from iron at the measurement step by electronic filtering. Time for data collection was typically 20 hours for a set of 457 measurements collected at room temperature in the range 0° < *θ* < 65° for equidistant *s* values [*s* = 4π(sin *θ*/*λ*)]. Treatment of the data was carried out according to a previously published procedure^56^ to allow the analysis of the radial distribution function (RDF) of the particles.

Magnetic measurements were performed on a Vibrating Sample Magnetometer (VSM, Quantum Device PPMS Evercool II). VSM studies were carried out on compact powder samples that were prepared and sealed under argon atmosphere.

For analysis by transmission electron microscopy (TEM), samples were prepared by the drop casting method on carbon coated 3.05 mm copper grids of 400 meshes from Pelanne instruments with a collodion film (thickness of around 20 to 50 nm). TEM images were recorded on a MET Jeol JEM 1011 and 1400 instrument, size distributions were acquired by measuring a minimum of 250 objects using the open source ImageJ software. Sizes are given as mean ± standard deviation according to a Gaussian fit of the corresponding size distribution.

For analysis by scanning electron microscopy (SEM), the NPs (in powder form) were deposited on carbon adhesive tapes. SEM images were recorded on a SEM JEOL 7800F.

Synthesis reactants, solvents and products were stored and manipulated in a glove-box under argon (<1 ppm H_2_O, <1 ppm O_2_). All syntheses were performed in Fisher–Porter or Schlenk tubes using classical argon-vacuum line techniques. Mesitylene (Fisher, >99%) was dried over sodium (Riedel-de Haën, 99%), distilled and degassed by 3 freeze–pump–thaw cycles. Anisole (Alfa Aesar, 99%) was degassed by 3 freeze–pump–thaw cycles then dried over molecular sieve (4 Å). Toluene and pentane (99%, VWR Prolabo) were purified through a MBraun SPS-800 purification machine and degassed by 3 freeze–pump–thaw cycles. [Fe[N(SiMe_3_)_2_]_2_]_2_ (Nanomeps), diphenylamine (DPA) (Sigma Aldrich, >99%), norbornene (Alfa Aesar, 99%) and HMDS (Sigma Aldrich, >99%) were used without any additional purification. H_2_ (<3.000 ppm H_2_O, <2.000 ppm O_2_, <1.000 ppm CO, <1.000 ppm CO_2_, <10.000 ppm N_2_) and Ar (<3 ppm H_2_O, <2 ppm O_2_) were purchased from Air Liquide. Poly-(2,6-dimethyl-1,4-phenyleneoxide) (PPO, average MW: 30 000) (Aldrich) was dried under vacuum over P_2_O_5_ (Sigma Aldrich, >98%) at 80 °C for one night before use.

The [Fe(NPh_2_)_2_]_2_ complex was prepared by metathesis between [Fe[N(SiMe_3_)_2_]_2_]_2_ and DPA (method 1, adapted from ref. 17) or directly from FeBr_2_ and LiNPh_2_ (method 2, adapted from ref. 18).

#### Method 1

The green iron complex [Fe[N(SiMe_3_)_2_]_2_]_2_ (376.6 mg, 0.5 mmol) was added to a colourless solution of DPA (846.1 mg, 5 mmol) in 20 mL of toluene. Immediately the colour of the solution changed to dark-red. This solution was stirred magnetically for 4 h at 110 °C, then dried under vacuum leading to a black red residue. In order to fully remove the by-product hexamethyldisilazane (HMDS), the residue was heated at 180 °C under vacuum for 3 minutes. It was then diluted in 20 mL of toluene, and after 2 days settling at room temperature, the supernatant was removed and the dark-red crystals obtained were washed with pentane (3 × 10 mL), dried and stored in the glove-box. Yield: 85%.

#### Method 2

A solution of DPA (338 mg, 2 mmol) in THF (15 mL) was put in an ice bath under stirring. *n*-BuLi (1.25 mL, 2 mmol) was added dropwise. The solution was left in the ice bath with stirring for 1 h. Then, FeBr_2_ (216 mg, 1 mmol) was added in 5 mL of THF. The solution was left under stirring overnight. The volatile compounds were removed under reduced pressure and a black residue was obtained. This residue was extracted with hot toluene (3 × 10 mL), the extracts were filtrated over a syringe filter (*Ø* 25 mm, 0.45 μm pores size) and crystallization was induced by adding a small quantity of pentane. Dark-red crystals were obtained which were treated as mentioned above. Yield: 16%.

ICP-OES and CHN analysis (experimental/calculated values in at%): Fe(13.5/14.2), C(71.3/73.5), H(5.0/5.15), N(7.0/7.15). ^1^H NMR (in C_6_D_12_): 7.23 (t, 16H), 7.05 (d, 16H), 6.91 (t, 8H); FTIR (ATR mode): 3100–2980 cm^−1^ (w), 1581 cm^−1^ and 1477 cm^−1^ (s), 1058 cm^−1^, (m), 750 cm^−1^ (s).

#### Sample 1

A green solution of [Fe[N(SiMe_3_)_2_]_2_]_2_ (376.62 mg, 0.5 mmol) in mesitylene (20 mL) was pressurized under 3 bars of H_2_ and heated for 48 h at 150 °C. The solution progressively turned black. After cooling to r.t., and evacuation of excess H_2_, the solution was evaporated to dryness and the black shiny product obtained was analysed by TEM, WAXS, VSM and ICP-OES.

#### Sample 2

A red solution of [Fe(NPh_2_)_2_]_2_ (392.3 mg, 0.6 mmol) in mesitylene (20 mL) was pressurized under 3 bars of H_2_ for 48 h at 150 °C upon which time a black material precipitated and deposited on the magnetic stirring bar. After cooling to r.t., and evacuation of excess H_2_, the colourless transparent supernatant was removed. The insoluble black material was washed with toluene (2 × 10 mL) and dried. The black powder obtained was analysed by ICP-OES, TEM, SEM, VSM and WAXS. The transparent colourless supernatant of the reaction was analysed by GC-MS.

#### Sample 3

[Fe(NPh_2_)_2_]_2_ (78.5 mg, 0.11 mmol) was added to a solution of PPO (223 mg) in anisole (10 mL). The red solution obtained was pressurized under 3 bars of H_2_ and heated during 48 h at 150 °C. The solution progressively turned black. The reacting medium was then treated as for sample 1. The black shiny product obtained was analysed by TEM, WAXS, VSM and ICP-OES.

#### Sample 4

[Fe[N(SiMe_3_)_2_]_2_]_2_ (75.32 mg, 0.11 mmol) was added to a solution of PPO (223 mg) in anisole (10 mL). The brown solution obtained was pressurized under 3 bars of H_2_ during 48 h at 150 °C. The solution progressively turned black. The reacting medium was then treated as for sample 1. The black shiny product obtained was analysed by TEM, WAXS, VSM and ICP-OES.

#### Sample 5

A green solution of [Fe[N(SiMe_3_)_2_]_2_]_2_ (376.62 mg, 0.5 mmol) in anisole (20 mL) was pressurized under 3 bars of H_2_ for 48 h at 150 °C. The solution progressively turned black. The reacting medium was then treated as for sample 1. The black shiny product obtained was analysed by TEM, WAXS, VSM and ICP-OES.

#### Sample 6

A red solution of [Fe(NPh_2_)_2_]_2_ (196.1 mg, 0.25 mmol) in anisole (10 mL) was pressurized under 3 bars of H_2_ for 48 h at 150 °C upon which time a black material precipitated and deposited on the magnetic stirring bar. The reacting medium was then treated as for sample 2. The black powder obtained was analysed by ICP-OES, WAXS, VSM and TEM.

#### Sample 7

[Fe(NPh_2_)_2_]_2_ (196.1 mg, 0.25 mmol) was added to a solution of HMDS (162.4 mg, 1 mmol) in mesitylene (10 mL). The red solution obtained was pressurized under 3 bars of H_2_ for 48 h at 150 °C upon which time a black material precipitated and deposited on the magnetic stirring bar. The reacting medium was then treated as for sample 2. The black powder obtained was analysed by ICP-OES, TEM, VSM and WAXS analysis. The transparent colourless supernatant of the reaction was analysed by GC-MS.

Hydride titration was performed following the protocol reported by Kelsen *et al.*^22^ On the crude colloidal solution of NPs in mesitylene, three freeze–pump cycles were performed in order to remove the dihydrogen possibly dissolved into the solvent. Then, 5 equivalents (with respect to the total number of iron atoms) of 2-norbornene were added. After stirring during 24 h at room temperature, the sample was filtered over silica and analyzed by GC-MS in order to determine the conversion of 2-norbornene into norbornane. The quantity of reactive surface hydrides was calculated from the conversion value. Knowing the proportion of surface atoms in each sample (60% for sample 1 and 63% for sample 5), the number of hydride per iron surface atom could be calculated: 0.80 H per surface iron atom for sample 1 and 0.16 H per surface iron atom for sample 5. Each titration was reproduced 3 times.

## Conclusions

In this work, a series of iron NPs was prepared from two different amido iron complexes ([Fe(N(SiMe_3_)_2_)_2_]_2_ and [Fe(NPh_2_)_2_]_2_) and fully characterized by TEM, SEM, VSM and WAXS to evidence their morphology, structure and magnetic properties. They divided into two categories: NPs of β-Mn structure and average sizes in the range 1.5–2 nm, and larger, ill-defined and aggregated NPs of bcc structure. The use of the new amido iron precursor, [Fe(NPh_2_)_2_]_2_, allowed shedding light on the stabilization of iron NPs, suggesting amido surface ligands could play a yet underestimated role. Thus, amido ligands might be interesting to investigate as a new class of ligands for iron based NPs in line with the work reported by Egeberg *et al.* on lithium pyridine stabilized iron NPs.^42^

Magnetic measurements, supported by DFT calculations, indicated that size reduction effects were easily counter balanced by surface coordination of amines and hydrides on the surface of the NPs. Hydride titration confirmed the theoretical prediction stating that these are the species responsible for the variations in magnetization observed. More precisely, the decrease in saturation magnetization value was clearly correlated to the increase in hydride surface coverage both theoretically and experimentally. These results suggest that the magnetization can be a good probe to investigate hydride surface coverage and might be a valuable indicator of the reactivity of iron NPs in hydrogenation reactions. In this work, hydrogenation of the phenyl rings of the diphenylamido ligand and of mesitylene was observed in some cases. Investigating whereas this was the result of molecular intermediate species, clusters or nanoparticles was out of the scope of this work but this observation opens new possible applications of the [Fe(NPh_2_)_2_]_2_ complex for the design of hydrogenation catalysts.

## Conflicts of interest

There are no conflicts to declare.

## Supplementary Material

NA-003-D1NA00258A-s001
