# Supplementary material for: Correlation between surface chemistry and magnetism in iron nanoparticles
Source: Nanoscale Adv. 2021 Jun 29;3(15):4471–81. doi: 10.1039/d1na00258a (PMC9419664; doi:10.1039/d1na00258a)
Supplement: NA-003-D1NA00258A-s001 [file NA-003-D1NA00258A-s001.pdf]

Electronic Supplementary Material (ESI) for Nanoscale Advances.
This journal is © The Royal Society of Chemistry 2021

Supporting Information for

Correlation between surface chemistry and magnetism in iron nanoparticles

Lorraine HAIM[1,2,3], François ROBERT[1,2], Laurent PERES[1,2], Pierre LECANTE[3], Karine PHILIPPOT[1,2], Romuald POTEAU*[4], Marc RESPAUD[3,4], Catherine AMIENS*[1,2]

[1] *CNRS, LCC (Laboratoire de Chimie de Coordination), 205, route de Narbonne, BP44099, F-31077 Toulouse Cedex 4, France*
[2] *Université de Toulouse, UPS, INPT, F-31077 Toulouse CEDEX 4, France*
[3]*CEMES (Centre d'Elaboration de Matériaux et d'Etudes Structurales), CNRS, 29 rue J. Marvig, F-31055 Toulouse, France*
[4]*LPCNO (Laboratoire de Physique et Chimie des Nano-Objets), UMR 5215 INSA, CNRS, UPS, 135 Avenue de Rangueil, F-31077 Toulouse Cedex-4, France*

1. List of the samples reported in this work 1
2. Potential by-products formed upon hydrogenation of the two iron precursors 2
3. WAXS analysis of the samples described in this work 3
4. Complementary data on sample 1 (TEM) 4
5. Complementary data on sample 2 (GC analysis, SEM and TEM images) 5
6. Complementary data on sample 7 (GC analysis) 7
7. Magnetic measurements 9
   - Sample 1 9
   - Sample 2 11
   - Sample 3 11
   - Sample 4 12
   - Sample 5 14
   - Sample 6 15
   - Sample 7 15
8. Supplementary Information associated to the theoretical part 16
   - 8.1. Bulk properties 16
   - 8.2. Various bare Fe_{91} models 17
   - 8.3. Magnetic properties 18
   - 8.4. Simulated interatomic distances histograms 19
   - 8.5. Ligand-protected Fe_{91} models 20

1. List of the samples reported in this work

Sample number	Precursor	Solvent	Stabilizing agent added
**1**	$[Fe[N(SiMe_3)_2]_2]_2$	Mesitylene	-
**2**	$[Fe(NPh_2)_2]_2$	Mesitylene	-
**3**	$[Fe(NPh_2)_2]_2$	Anisole	PPO
**4**	$[Fe[N(SiMe_3)_2]_2]_2$	Anisole	PPO
**5**	$[Fe[N(SiMe_3)_2]_2]_2$	Anisole	-
**6**	$[Fe(NPh_2)_2]_2$	Anisole	-
**7**	$[Fe(NPh_2)_2]_2$	Mesitylene	HMDS

Table S1: Reaction conditions for each sample. Duration (48h), temperature (150°C) and hydrogen pressure (3 bar) were identical for all samples. PPO = polydimethylphenylene oxide, HMDS = hexamethyldisilazane

2. Potential by-products formed upon hydrogenation of the two iron precursors

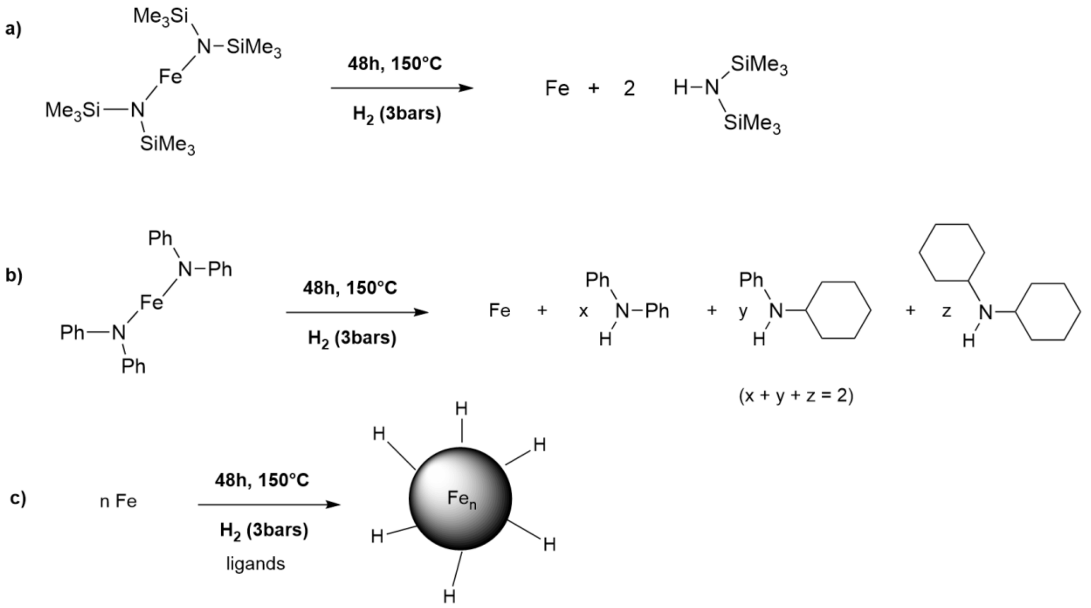

Scheme S1: tentative schemes for the hydrogenation of a) $[Fe[N(SiMe_3)_2]_2]_2$ and b) $[Fe(NPh_2)_2]_2$ complexes showing the potential by-products formed that could play the role of ligands at the surface. The Fe atoms thus generated will nucleate and form the NPs. Hydrogen potentially dissociates (more or less easily depending on the ligands present) into hydrides as in c) ; ligands are not drawn at the surface of the NP in this generic scheme for clarity. It is noteworthy that all steps a (or b) and c can occur simultaneously and that the amines can also dissociate at the surface (activation of the NH bond) leading to surface amido ligands as described in Figures SI19.

3. WAXS analysis of the samples described in this work

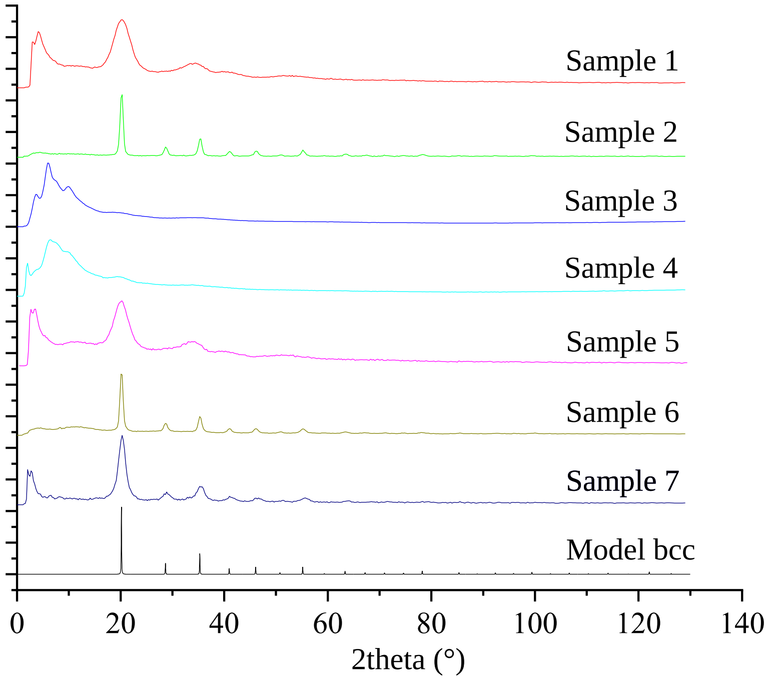

Figure S1: WAXS diagrams in reciprocal space in comparison with the bcc-Fe data (PDF-01-071-4648).

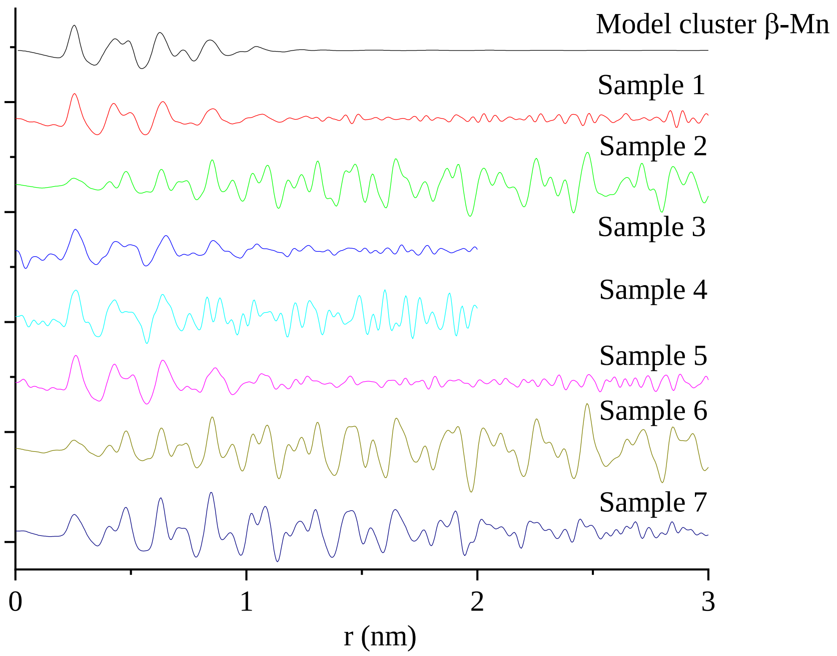

Figure S2: Radial Distribution Functions in comparison with that of a β-Mn reference cluster of 101 atoms.

4. Complementary data on sample 1 (TEM)

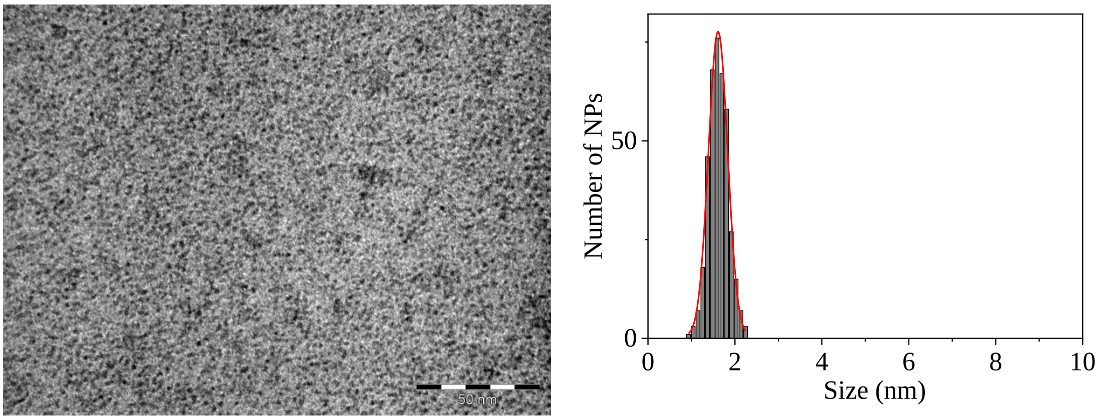

Figure S3: TEM images of **sample 1** and corresponding size distribution (scale bar 50nm)

5. Complementary data on sample 2 (GC analysis, SEM and TEM images)

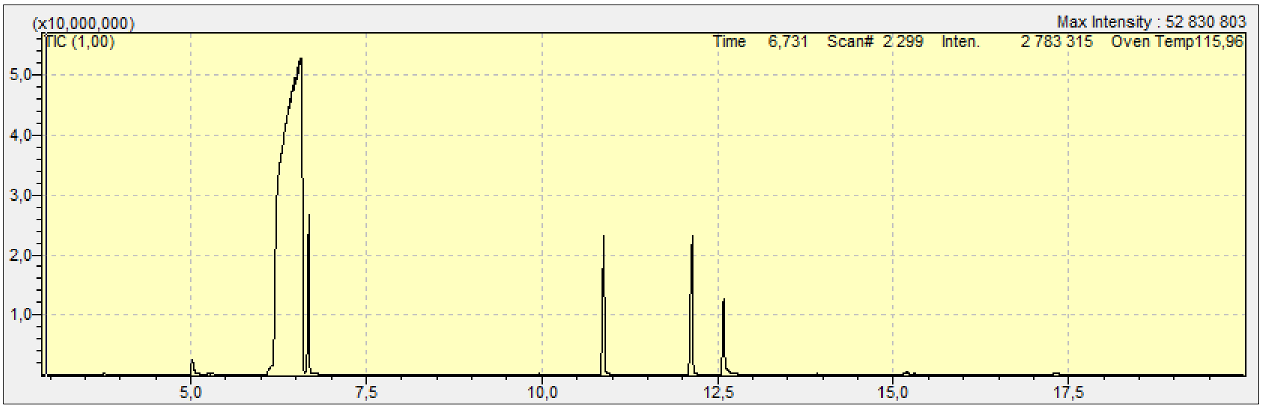

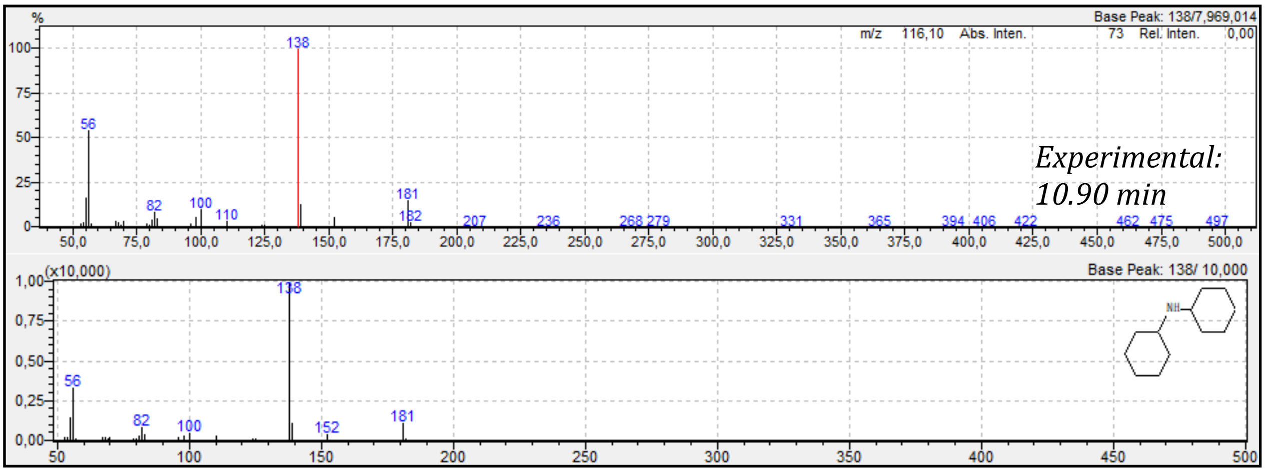

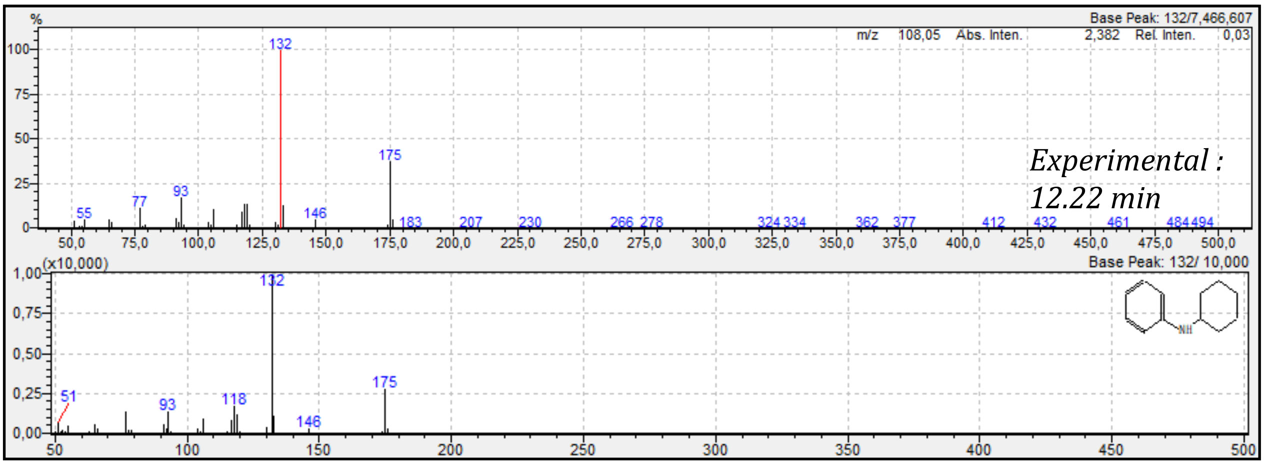

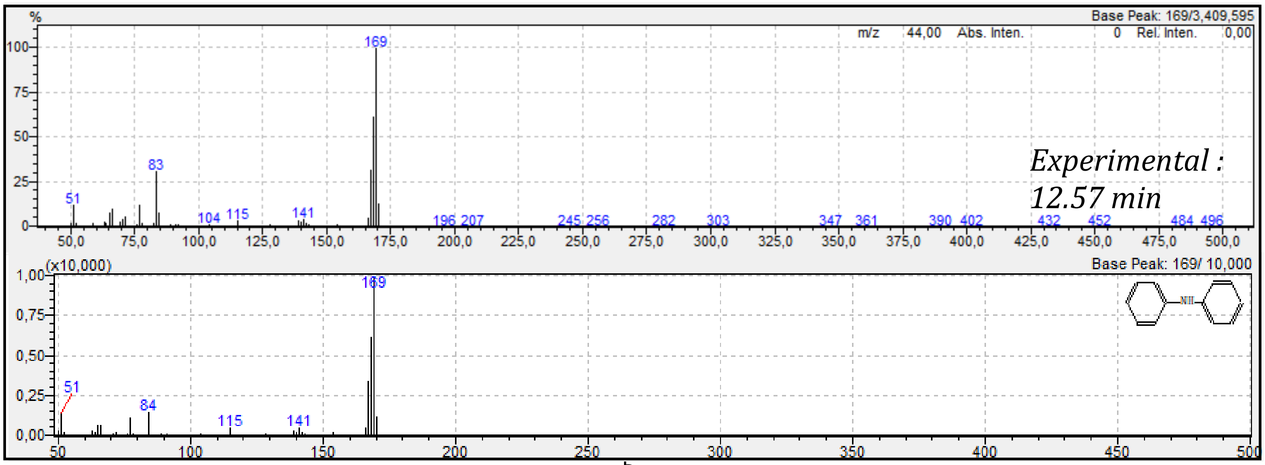

Figure S4: from top to bottom: GC analysis of the supernatant and mass spectra of the product corresponding to retention time of respectively 10.90, 12.22 and 12.57 min and comparison to the reference mass spectra from the NIST data base

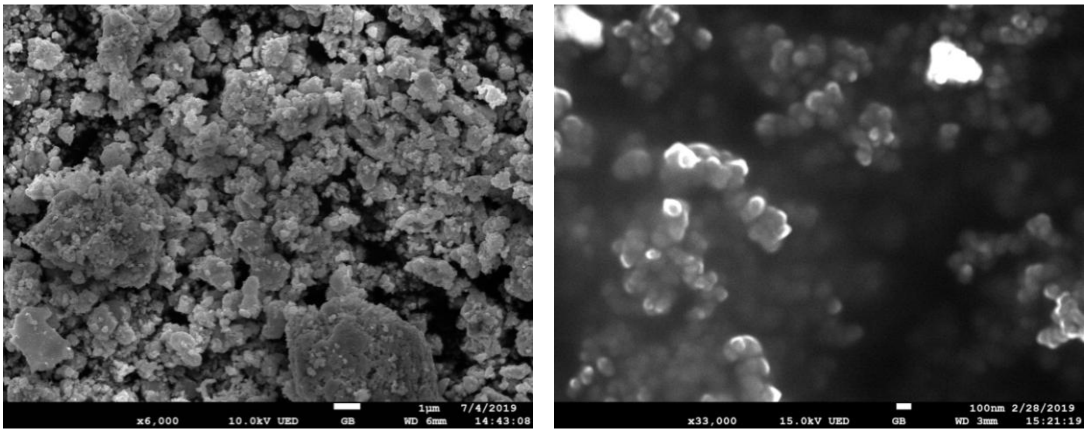

Figure S5 : SEM images of **sample 2** (left scale bar 1 µm ; right scale bar 100 nm)

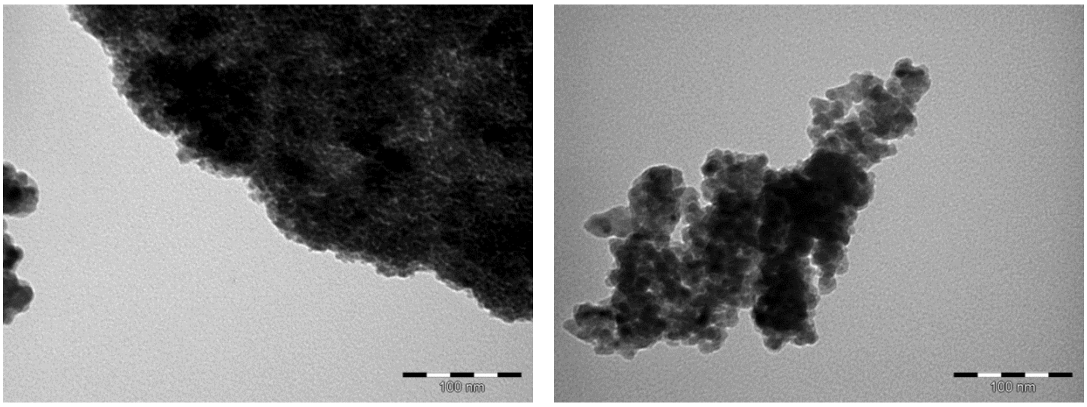

Figure S6: TEM images of **sample 2** (scale bar 100nm)

6. Complementary data on sample 7 (GC analysis)

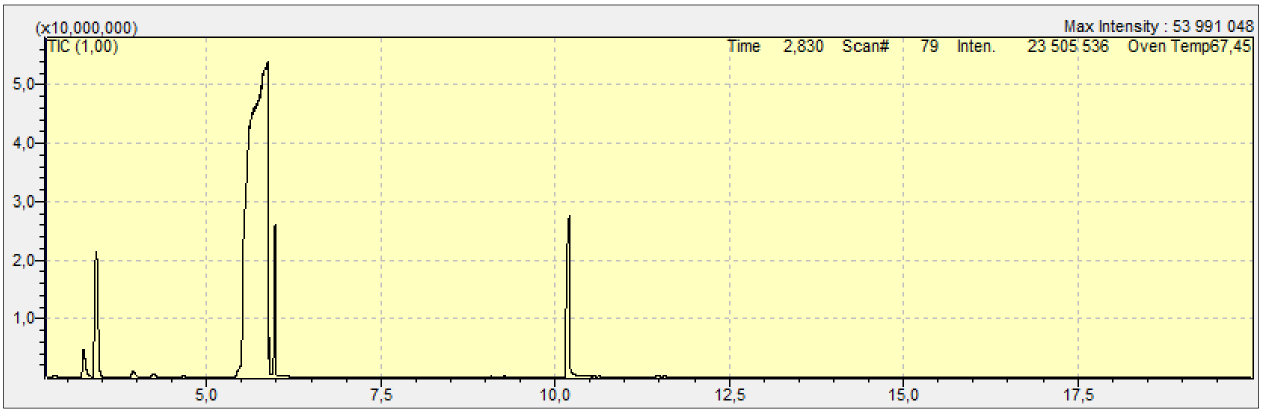

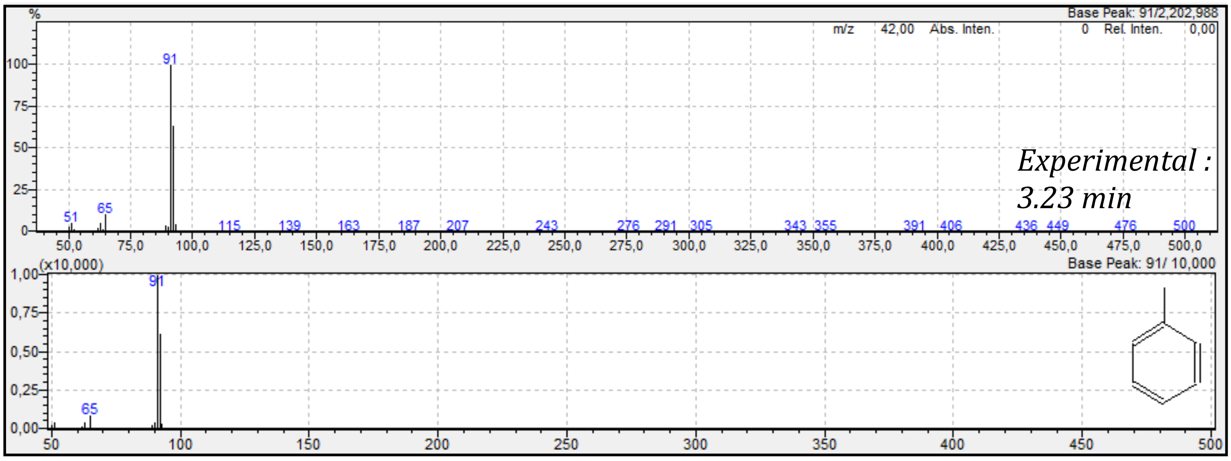

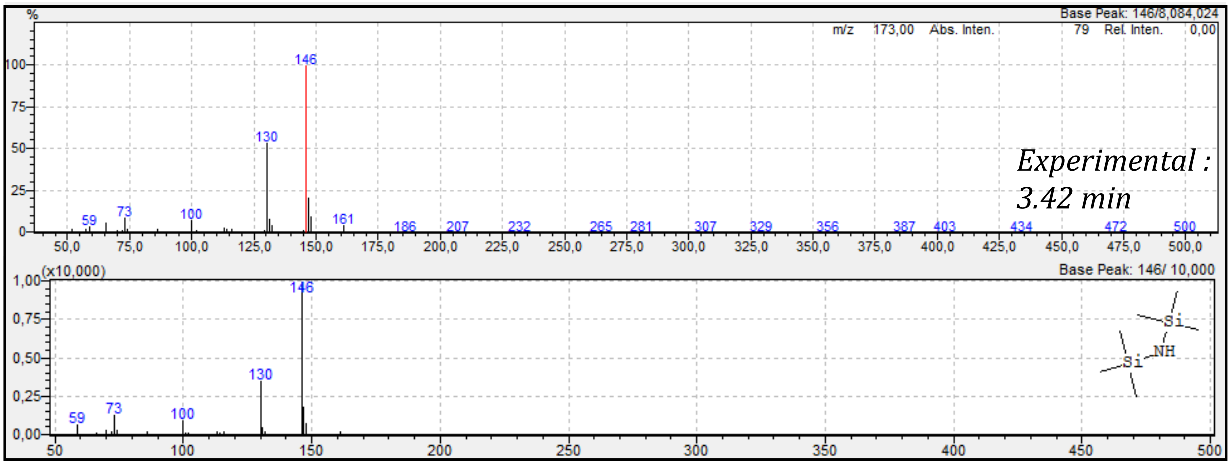

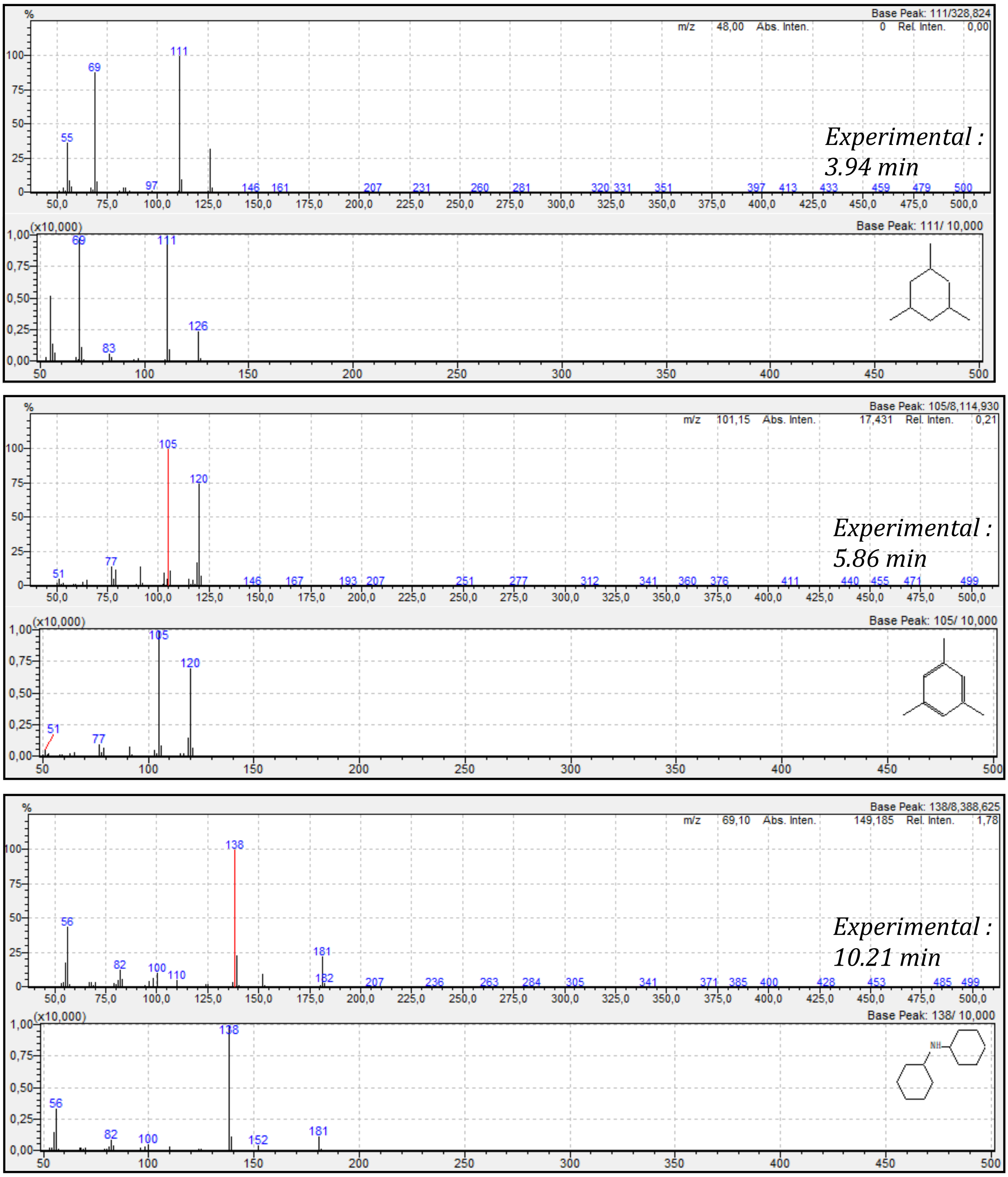

Figure S7: From top to bottom : GC analysis of the supernatant of **sample 7** and mass spectra of the products corresponding to retention times of respectively 3.23, 3,42, 3,94, 5,86 and 10.21 min and comparison to the reference mass spectra from the NIST data base

7. Magnetic measurements

Magnetic measurements were performed with a Vibrating Sample Magnetometer (VSM, Quantum Device PPMS Evercool II). VSM studies were carried out on compact powder samples that were prepared and sealed under argon atmosphere. Magnetization measurements were normalized by the Fe mass within the measured sample, determined by inductively coupled plasma-optical emission spectrometry (ICP-OES). Magnetization values (M) are thus given in $A.m^2/kg_{Fe}$. The average magnetic moment per Fe atom (μ_{Fe}) can be calculated as follows: $\mu_{Fe} = M \times M_{mol} / N_a / \mu_B$, where M_{mol} is the molar mass of Fe (55.845 10^{-3} kg/mol), N_a = 6.022 10^{23} atoms/mol is the Avogadro number, and μ_B = 9.27 10^{-24} $J.T^{-1}$. The magnetic moment per atom can be directly compared with theoretical data. We estimate that the total error is around 5% as indicated in Table 1.

For the low temperature measurements, magnetization curves were recorded after a field cooling (FC) from 300K down to 2.5 or 5K, under a magnetic field of 5T. These hysteresis loops were compared with those recorded after a zero field cooling (ZFC) procedure (cooling from r.t. down to the low temperature in the absence of magnetic field). This procedure should unveil exchange phenomena which occur in the case of ferromagnetic metallic Fe / ferrimagnetic Fe oxide interface. Such an interface may induce an asymmetric hysteresis loop at low temperature, when measured after a FC procedure. We didn't observe such phenomenon on any of the samples studied, which allowed concluding that the NPs are metallic.

Data were analyzed following the procedures described in reference ([1]) using the fitting procedure of the low temperature ZFCFC, or by fitting the isothermal magnetization curves in the superparamagnetic regime. Either procedure allowed estimating the magnetic NP size and the effective magnetic anisotropy.

Sample 1

Magnetic measurements evidenced a saturation magnetization reaching 203 $A.m^2.kg_{Fe}^{-1}$ at 5K after FC (Figure S8) and a coercive field of 19mT at 5K. The NPs displayed a superparamagnetic behaviour with a blocking temperature below 5K. Analysis of the zero field cooled / field cooled (ZFC/FC) magnetization recorded at 10mT evidenced a Curie Weiss behaviour, with θ = -3K indicative of weak dipolar interactions. The isothermal magnetization curves were analyzed with modified Langevin functions, using a NP diameter centered at 1.5nm, and an effective magnetic anisotropy K_{eff} = 6.3 $10^5 J.m^{-3}$ (See Table 1, and Figure S8).

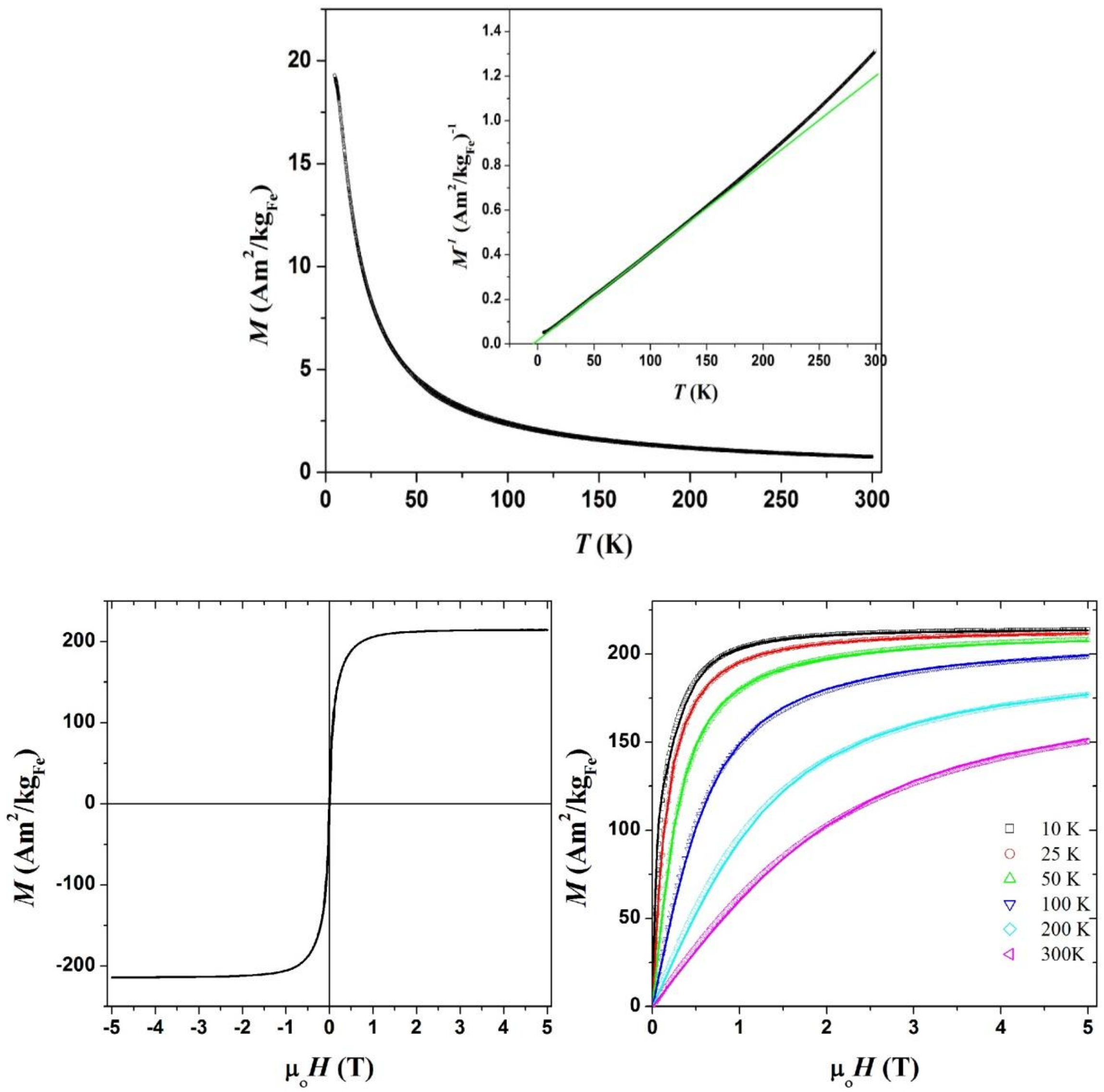

Figure S8: Magnetic investigation of **sample 1**. Top: ZFC/FC curves recorded at 10mT (in black). Inset showing the variation of the inverse of the magnetization versus temperature. In green, fit of the low temperature data with a simple Curie-Weiss law. Bottom left: hysteresis loops measured at 5K after FC; Bottom right: magnetization curves recorded at 10, 25, 50, 100, 200 and 300K (dots), showing a superparamagnetic behaviour, and fit of the data (solid lines).

Sample 2

The main features of the magnetic properties are shown in Figure S9 and reported in Table 1. For this nanostructured iron powder of bcc structure, the hysteresis cycle recorded at 5K after a FC displayed only a very small coercive field (13mT) and a magnetization of 205 A.m^2.kg$_{Fe}^{-1}$ at 5T. Note that this hysteresis loops is symmetric, which confirms the absence of Fe oxide domains.

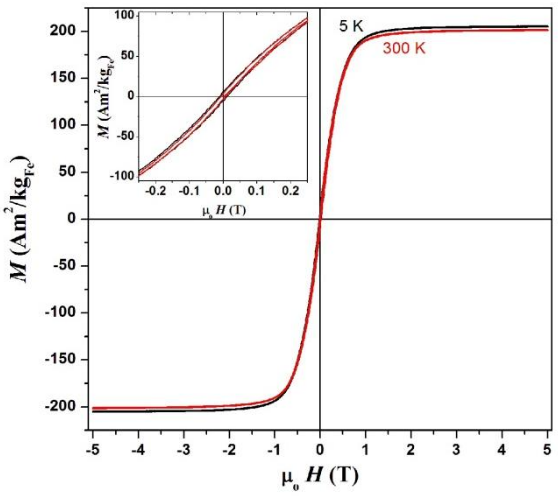

Figure S9 : Hysteresis cycle recorded from **sample 2** (inset: enlargement showing the limited coercivity of the sample). Low temperature data were collected after a field cooling from 300K down to 5K under a magnetic field of 5T.

Sample 3

Magnetic measurements evidenced a superparamagnetic behaviour (Figure 10) with a blocking temperature at 12.2K. The variation of the inverse of the magnetization against the temperature followed a straight line crossing the x axis at -4.5K. This value is indicative of limited magnetic dipolar couplings between the nanoparticles, in agreement with their good dispersion in the polymer matrix. A good fit of the ZFC/FC curve could be achieved taking into account a narrow log normal size distribution centred on a diameter of 2.2 ± 0.16 nm. The value of the effective magnetic anisotropy constant was extracted from this fit : K_{eff} = 3.8 10^5 J.m^{-3}. The hysteresis cycle (Figure S10) recorded at 2.5K after a FC procedure from 300K in a magnetic field of 5T was symmetrical and showed a relatively large coercive field (103mT) for Fe nanoparticles. This hysteresis loop exactly superimposed on the one recorded after a ZFC sequence, thus confirming the metallic ferromagnetic character of the iron nanoparticles. After correction of the diamagnetic contribution from the polymer matrix, the value of the saturation magnetization measured at 5T (214 A.m^2.kg$_{Fe}^{-1}$) is very close to that of bulk iron (2.22μ$_B$ = 222 A.m^2.kg$_{Fe}^{-1}$ at 5K).

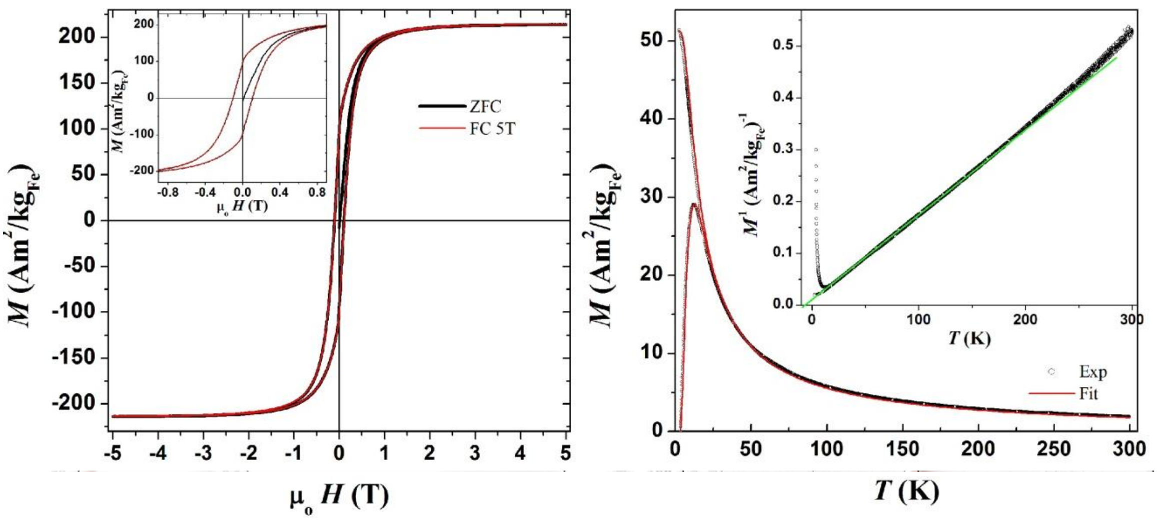

Figure S10: Magnetic investigations on **sample 3**. Left: hysteresis loops measured at 2.5K according to ZFC (black) and FC from r. t. under 5T (red) protocols. Right: ZFC/FC curves recorded at 10mT (in black) and fit of the data (in red). Inset showing the variation of the inverse of the magnetization versus temperature. In green, fit of the low temperature data with a simple Curie-Weiss law.

Sample 4

Magnetic measurements (Table 1) according to a ZFC/FC procedure evidenced a superparamagnetic behaviour (Figure S11) with a blocking temperature at 11.3 K. The variation of the inverse of the magnetization against the temperature followed a straight line crossing the *x* axis at 7K indicative of limited magnetic dipolar couplings between the nanoparticles in agreement with the good dispersion of the nanoparticles in the polymer matrix as for **sample 3**. A good fit of the ZFC/FC curve could be achieved taking into account a narrow log normal size distribution centred on a diameter of 1.7 ± 0.15 nm, leading to a K_{eff} value of 7.9 10^5 J.m^{-3}. The hysteresis cycle recorded at 2.5K was symmetrical and showed a small coercive field (37mT). Hysteresis loops recorded after ZFC and FC procedures superimposed, thus confirming the metallic character of the NPs. After correction of the diamagnetic contribution from the polymer matrix, the value of the saturation magnetization could be determined: 280 A.m^2.$\mathrm{kg}_{\mathrm{Fe}}^{-1}$ (at 5T). This value is far above that of bulk iron.

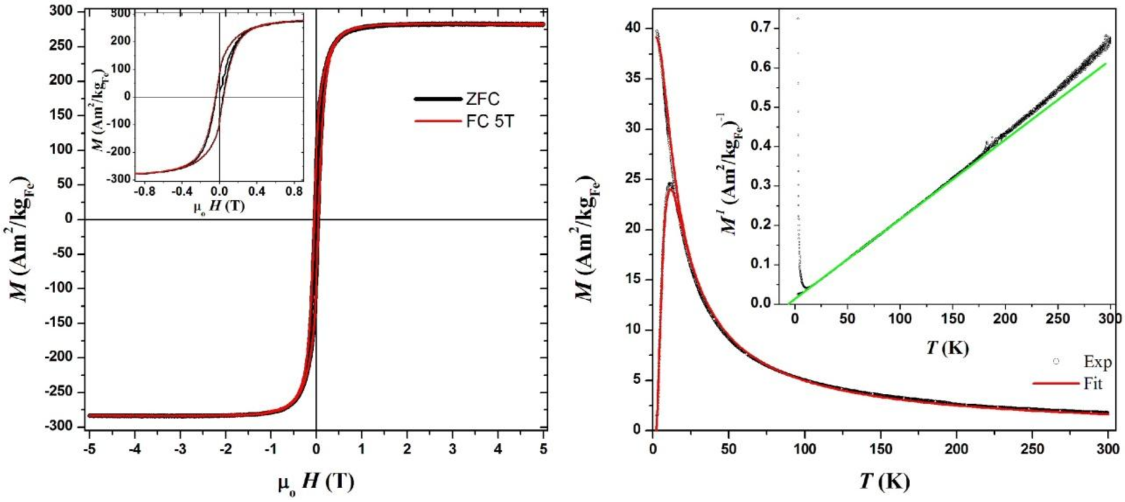

Figure S11: Magnetic investigation of **sample 4**. Left: hysteresis loops measured at 2.5K according to ZFC (in black) and FC from r. t. under 5T (in red) protocols. Right: ZFC/FC curves recorded at 10mT (in black) and fit of the data (in red). Inset showing the variation of the inverse of the magnetization versus temperature. In green, fit of the low temperature data with a simple Curie-Weiss law.

Sample 5

The low temperature hysteresis loop confirmed the metallic character of the NPs. The saturation magnetization could be determined at 249 A.m^2.kg$_{Fe}^{-1}$ (at 5T). This value is above the bulk iron. Magnetic measurements (Table 1) according to a ZFC/FC procedure evidenced a superparamagnetic behaviour (Figure S12) with a blocking temperature at 9.1 K. The variation of the inverse of the magnetization against the temperature followed a straight line crossing the x axis at -12 K indicative of some dipolar magnetic couplings between the nanoparticles. Dipolar interactions are here too strong to apply the ZFCFC fitting procedure. The fitting of the isothermal magnetization curves in the superparamagnetic regime allowed us to determine the average particle size 1.7 nm and the effective magnetic anisotropy K_{eff} = 8 10^5 J.m^{-3}.

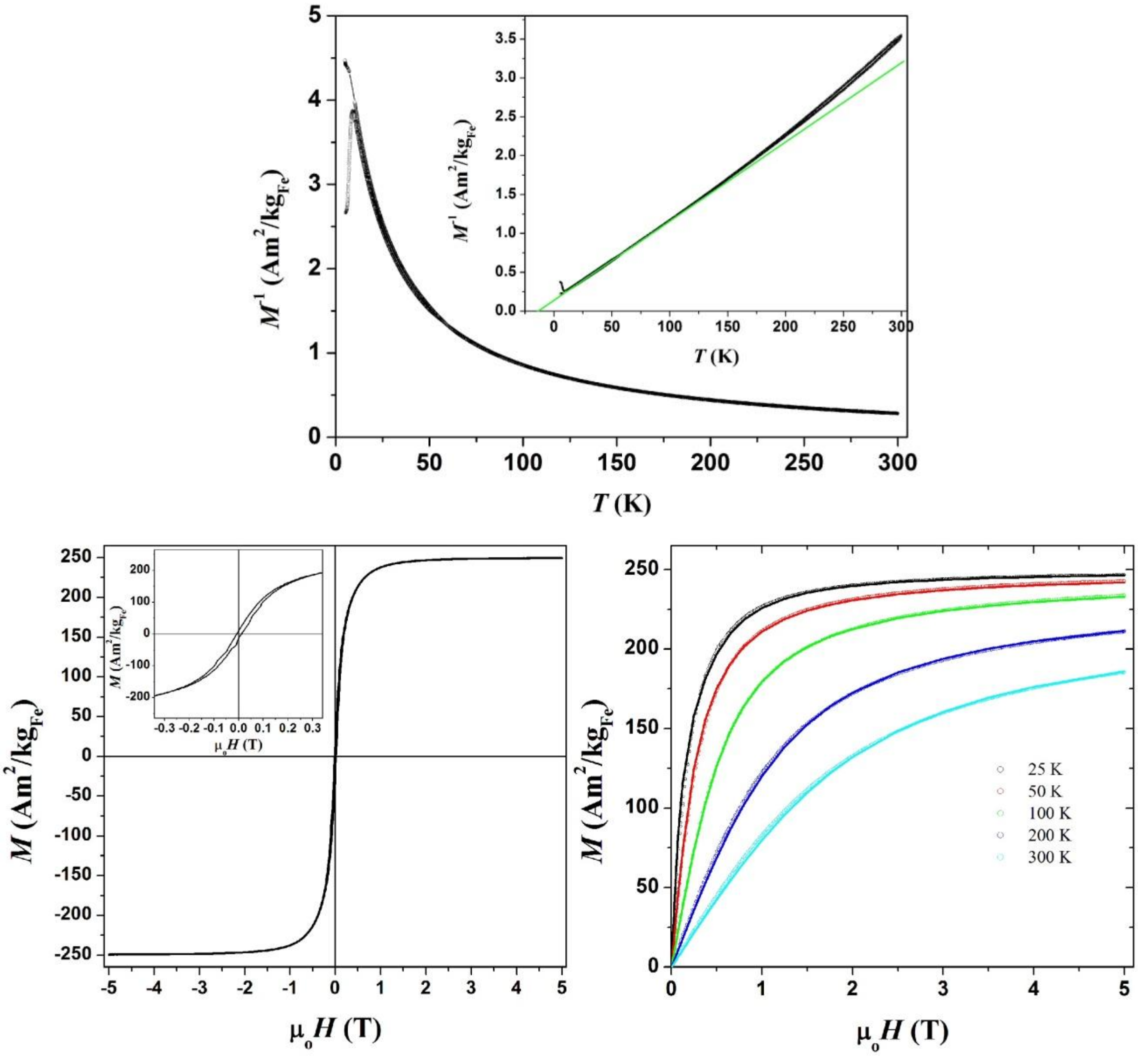

Figure S12 : Magnetic investigation of **sample 5**. Top: ZFC/FC curves recorded at 2.5mT (in black). Inset showing the variation of the inverse of the magnetization versus temperature. In green, fit of the low temperature data with a simple Curie-Weiss law. Bottom Left: hysteresis loops measured at 5K measured after FC. Bottom right: magnetization curves recorded at 25, 50, 100, 200 and 300K (dots), showing a superparamagnetic behaviour, and fit of the data (solid lines).

Sample 6

The magnetic properties were typical of metallic Fe (symmetric hysteresis loops) with a saturation magnetization of 210 A.m^2.kg$_{Fe}^{-1}$, a value close to that of bulk iron as reported in Table 1 and Figure S13. Note that this nanomaterial displayed a hysteretic behaviour at r. t. as a result of the high packing density.

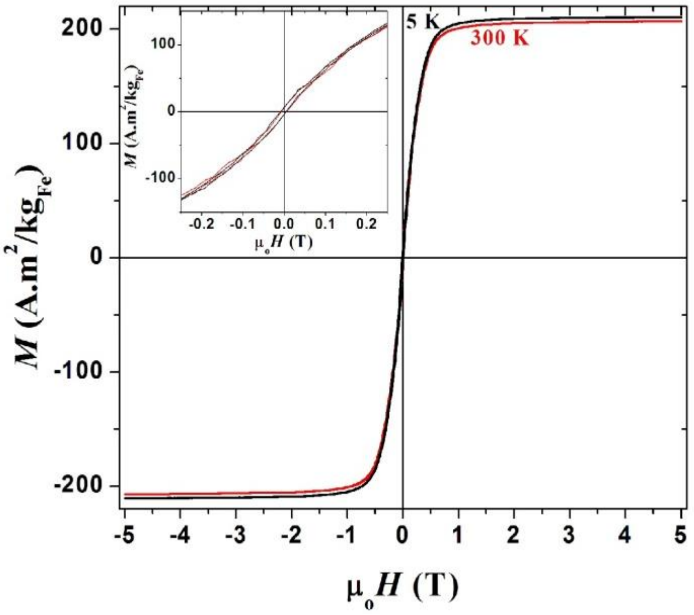

Figure S13 : Magnetic investigation of **sample 6**. Hysteresis loops measured at 5K (after FC) and 300K.

Sample 7

The magnetization measured at 5K (212 A.m^2.kg$_{Fe}^{-1}$) was very near the bulk one (see Figure S14). Note that the hysteretic behaviour vanishes at r. t..

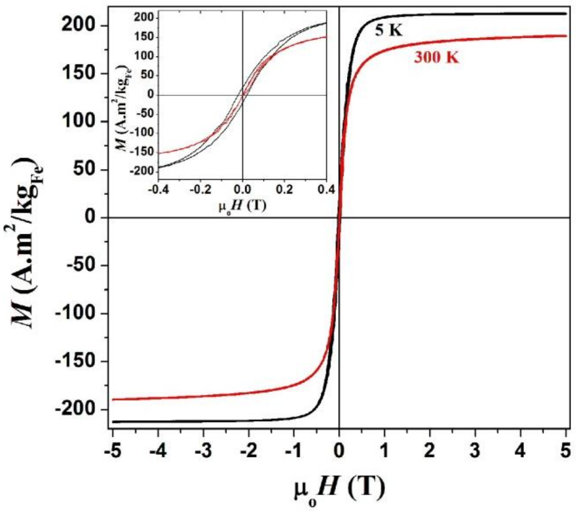

Figure S14: Magnetic investigation of **sample 7**. Hysteresis loops measured at 5K (after FC) and 300K.

8. Supplementary Information associated to the theoretical part

8.1. Bulk properties

Table S2: Comparison of bcc bulk solid properties with experimental data and other theoretical studies. The *β*-Mn phase does not exist for bulk iron. It has been optimized theoretically for the purpose of comparison with the experimental polytetrahedral nanoclusters reported and theoretically investigated in the present paper.

	**bcc**	**β-Mn type**
unit cell	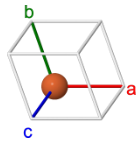 1 atom<br>This work: $a = b = c =$ 2.83 Å<br>exp[2]: 2.87 Å<br>PBEsol functional[3]: 2.786 Å<br>M06-L functional[3]: 2.856 Å	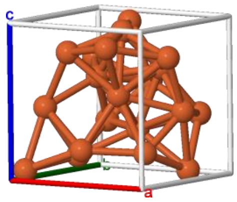 20 atoms<br>$a = b = c =$ 6.2 Å
cohesive energy, E_{coh} / eV	This work: 4.85<br>exp: 4.28<br>PW functional[4]: 4.78<br>PBEsol functional[3]: 5.67<br>M06-L functional[3]: 5.03	This work: 4.72
magnetic moment per atom, μ / μ_B	This work: 2.21<br>exp[2]: 2.22	This work: 2.40

magnetization colour maps, defined w.r.t. the PBE bulk solid value

(atomic values in μ_B)

white: 2.21 μ_B

blue: > 2.21 μ_B

red: < 2.21 μ_B

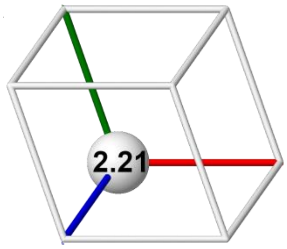

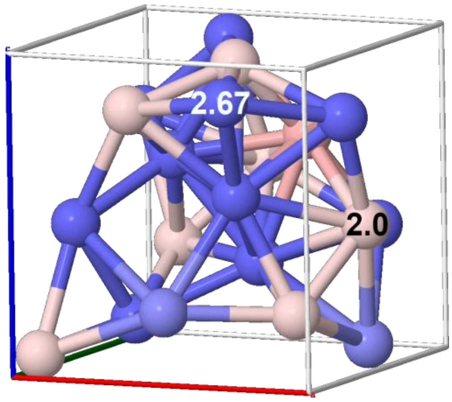

8.2. Various bare Fe_{91} models

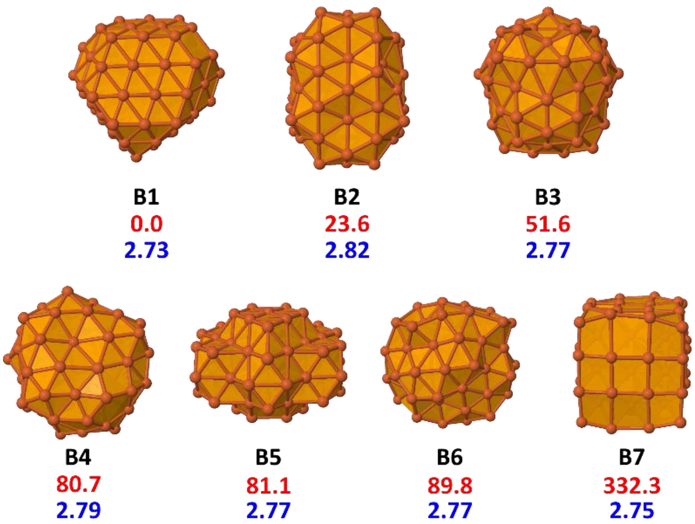

Figure S15. Bare Fe_{91} models considered in this study. B1: size-adapted rhombic dodecahedron; B2: Finnis-Sinclair optimal polytetrahedral structure[5]; B3: spherical β-W-based isomer; B4: spherical α-Mn-based isomer; B5: oblate bcc spheroid; B6: spherical β-Mn isomer; B7: bcc cube[6] (this isomer studied in a previous study has determined the choice of 91-atoms isomers). Second line (red values): energies (in kcal/mol) given w.r.t. B1. Third line (in blue): magnetic moments per atom (in μ_B units).

8.3. Magnetic properties

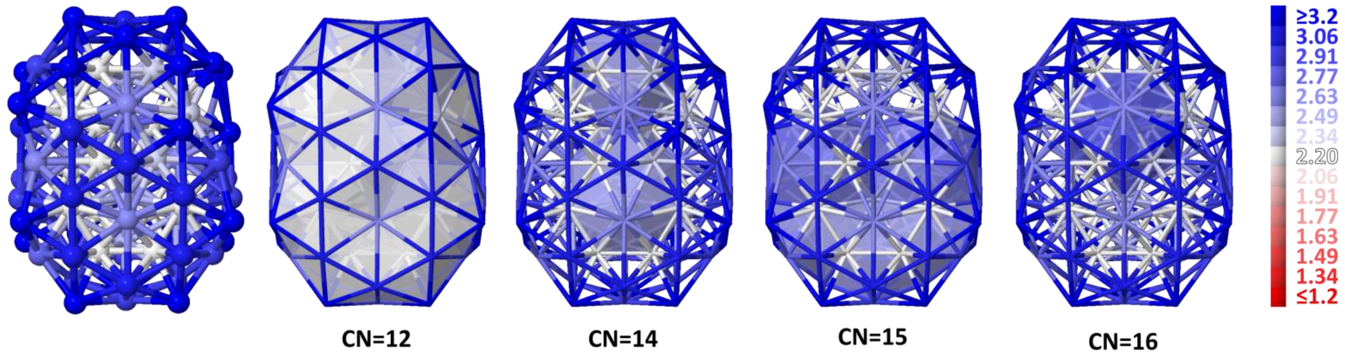

Figure S16. Bare Fe_{91} cluster: coordination polyhedra, coloured with the magnetization scale used all throughout the theroretical part of this paper. CN stands for coordination number.

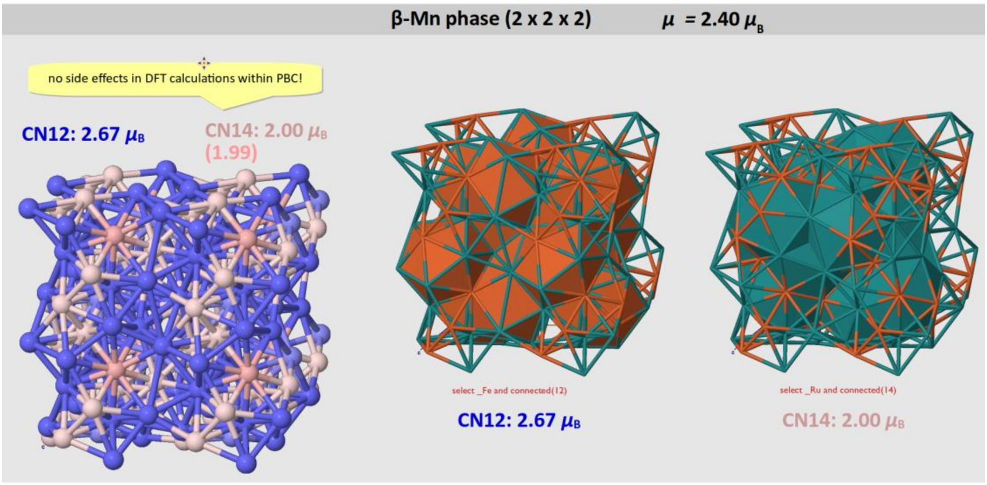

Figure S17. β-Mn phase of bulk iron: coordination polyhedra (same colour scale as in Figure S16). On the contrary to the bare Fe_{91} case, the lower the CN, the higher the atomic magnetic moment. It can be understood in terms of interatomic distances between the central atom and its neighbours (see Figure S18).

8.4. Simulated interatomic distances histograms

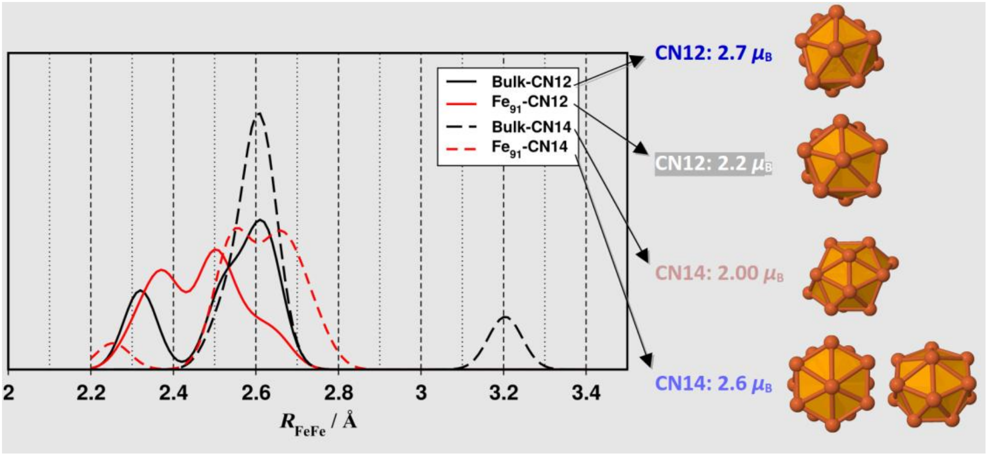

Figure S18. Histogram of the interatomic distances between the central atom and its nearest neighbours in the calculated *β*-Mn-type phase of iron and in the Fe_{91} cluster. The shapes of coordination polyhedra are also shown. Atoms around CN=12 iron atoms exhibit the same distorted icosahedron structure. The higher magnetic moment in the bulk is probably due to a lower packing efficiency, as evidenced by the histogram (plain black line, second peak at 2.6 Å *vs*. plain red line, second peak at 2.5 Å). The CN=14 coordination polyhedra differ in Fe_{91} and in the bulk. And the difference in the magnetic moment seems also related to the packing efficiency, which is weaker for the bulk (dashed black line) than for the cluster (dashed red line), in line with a higher magnetization in the bulk for such site.

8.5. Ligand-protected Fe_{91} models

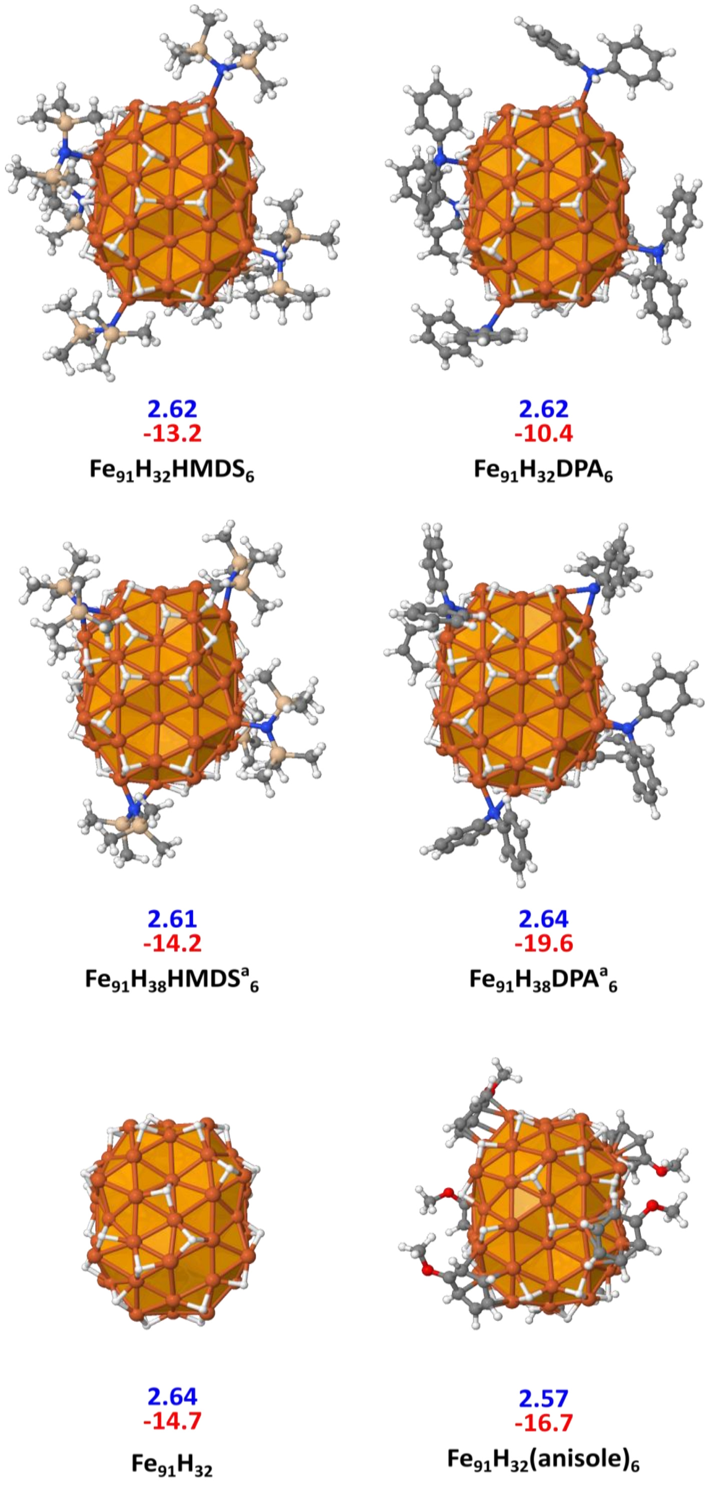

Figure S19. Fe_{91} model with τ_{surf} = 0.5 and 6 surface stabilizers. First line: magnetic moment per iron atom (in μ_B) ; second line: adsorption energy (in kcal/mol). Adsorption energies for amines correspond to the reaction: 6 Amine + $Fe_{91}H_{32}$ → $Fe_{91}H_{32}(Amine)_6$, whilst for amides it has to be considered as dissociative adsorption energies of amines on the iron NP surface, *i.e.*: 6 Amine + $Fe_{91}H_{32}$ → $Fe_{91}H_{38}(Amide)_6$. (the ^{a} exponent in the chemical formula stands for amide). The dissociative adsorption energy of H_2 per surface hydride is given for $Fe_{91}H_{32}$ (see computational details)

Bibliography

1 O. Margeat, M. Respaud, C. Amiens, P. Lecante and B. Chaudret, *Beilstein J. Nanotechnol.*, 2010, **1**, 108–118.
2 Charles Kittel, *Introduction to Solid State Physics*, John Wiley & Sons, 8th Edition., 2005.
3 G.-X. Zhang, A. M. Reilly, A. Tkatchenko and M. Scheffler, *New J. Phys.*, 2018, **20**, 063020.
4 P. H. T. Philipsen and E. J. Baerends, *Phys. Rev. B*, 1996, **54**, 5326–5333.
5 J. A. Elliott, Y. Shibuta and D. J. Wales, *Philos. Mag.*, 2009, **89**, 3311–3332.
6 G. Fischer, R. Poteau, S. Lachaize and I. C. Gerber, *Langmuir*, 2014, **30**, 11670–11680.
